# Radiomic profiles improve prognostication and reveal targets for therapy in cervical cancer

**DOI:** 10.1038/s41598-024-61271-4

**Published:** 2024-05-17

**Authors:** Mari Kyllesø Halle, Erlend Hodneland, Kari S. Wagner-Larsen, Njål G. Lura, Kristine E. Fasmer, Hege F. Berg, Tomasz Stokowy, Aashish Srivastava, David Forsse, Erling A. Hoivik, Kathrine Woie, Bjørn I. Bertelsen, Camilla Krakstad, Ingfrid S. Haldorsen

**Affiliations:** 1 Centre for Cancer Biomarkers, Department of Clinical Science, University of Bergen, Bergen, Norway; 2 Department of Obstetrics and Gynecology, Haukeland University Hospital, Bergen, Norway; 3 Mohn Medical Imaging and Visualization Centre, Department of Radiology, Haukeland University Hospital, Bergen, Norway; 4 Department of Mathematics, University of Bergen, Bergen, Norway; 5 Section of Radiology, Department of Clinical Medicine, University of Bergen, Bergen, Norway; 6 Genomics Core Facility, Department of Clinical Science, University of Bergen, Bergen, Norway; 7 Section of Bioinformatics, Clinical Laboratory, Haukeland University Hospital, Bergen, Norway; 8 Department of Pathology, Haukeland University Hospital, Bergen, Norway

**Keywords:** Uterine cervical neoplasms, Magnetic resonance imaging, Imaging genomics, Molecular targeted treatment, Cluster analysis, Cancer, Cell biology, Computational biology and bioinformatics, Genetics, Molecular biology, Biomarkers, Oncology, Mathematics and computing

## Abstract

Cervical cancer (CC) is a major global health problem with 570,000 new cases and 266,000 deaths annually. Prognosis is poor for advanced stage disease, and few effective treatments exist. Preoperative diagnostic imaging is common in high-income countries and MRI measured tumor size routinely guides treatment allocation of cervical cancer patients. Recently, the role of MRI radiomics has been recognized. However, its potential to independently predict survival and treatment response requires further clarification. This retrospective cohort study demonstrates how non-invasive, preoperative, MRI radiomic profiling may improve prognostication and tailoring of treatments and follow-ups for cervical cancer patients. By unsupervised clustering based on 293 radiomic features from 132 patients, we identify three distinct clusters comprising patients with significantly different risk profiles, also when adjusting for FIGO stage and age. By linking their radiomic profiles to genomic alterations, we identify putative treatment targets for the different patient clusters (e.g., immunotherapy, CDK4/6 and YAP-TEAD inhibitors and p53 pathway targeting treatments).

## Introduction

Uterine cervical cancer (CC) is the fourth most common cancer type in women globally^1^ and the second most common cancer in women aged 15 to 44 years^2^. Cervical cancer survival is closely linked to tumor extent at primary diagnosis^3^ which has traditionally been based on clinical staging according to the International Federation of Gynecology and Obstetrics (FIGO) 2009 classification^4^. However, in 2018, the FIGO Gynecologic Oncology Committee revised their guidelines allowing staging based on imaging- and pathological findings, when available^5^. Pelvic magnetic resonance imaging (MRI) is the preferred imaging modality for local and regional staging of macroscopically visible cervical cancer^6^. MRI can accurately assess important prognostic indicators such as primary tumor size, tumor invasion to the parametrium or pelvic sidewall, and enlarged pelvic lymph nodes^7,8^. As MRI is an established part of the diagnostic workup in cervical cancers in most high-income countries^9^, MRI radiomic tumor profiling has become possible. Radiomics utilizes high-throughput feature extraction methods in images to unravel tumor patterns and -characteristics that are invisible to the human eye^10^. The derived tumor radiomic signatures may, in addition to standard imaging findings and staging information, potentially aid in risk classification and personalizing patient treatment.

Although early detected disease is predominantly curable with surgery, treatment for late-stage or recurrent cervical cancer is heavily invasive, inferring severe short- and long-term side-effects and associates with poor survival. Hence, treatment schemes for late-stage and recurrent disease need to be refined and personalized to minimize side-effects and prolong survival. Personalized medicine in cancer treatments aims to identify and individualize therapy based on the tumor’s genomic- or molecular aberrations using biomarkers to select patients to the most beneficial therapy. Yet, large-scale diagnostic profiling of cancer genomes detecting actionable aberrations is currently unavailable, due to high costs, considerable time burden, and high complexity of data analyses and -interpretation^11^. Furthermore, genomic- and molecular characterization is hampered by representing only a fraction of the tumor and by biopsies being prone to selection biases. Whole-volume radiomic profiling allows assessment of the entire primary tumor volume that may contribute to the prediction model. Radiogenomics combines genomic- and radiomic data^12^ and may play an important role in identifying imaging surrogates that correlate with genomic profiles, thus serving as a substitute for genomic profiling^13^. The aim of this study was to perform a comprehensive radiogenomic characterization of primary tumors in cervical cancer and assess whether whole-volume MRI radiomic profiles can be linked to clinical phenotypes, patient outcome and potential molecular- or genomic therapeutic targets in CC.

## Materials and methods

### Patient cohort, biospecimen collection and ethical statements

**Figure 1:**
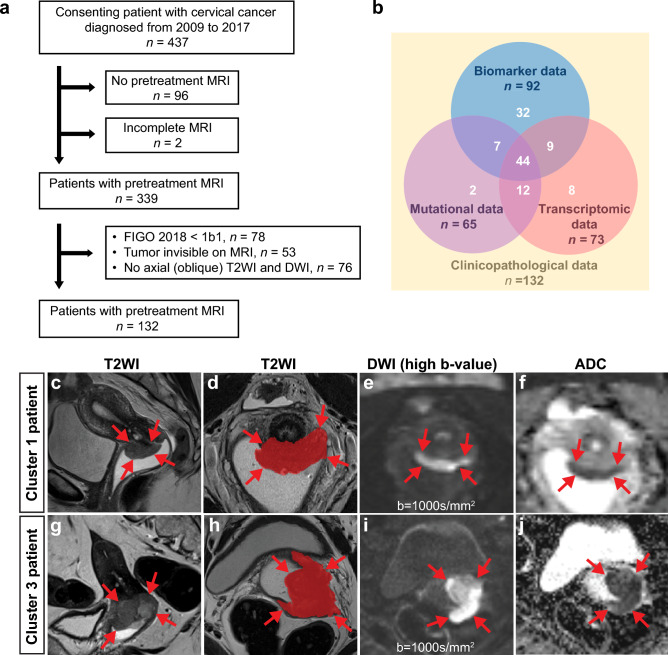
Exclusion criteria, overlapping data in study cohort and examples of segmented tumors on MRI. (**a**) The study cohort is established from a patient cohort of consenting patients (participation rate > 95%) with histopathologically confirmed cervical cancer diagnosed from 2009 to 2017 at Haukeland University Hospital (Bergen, Norway). The included patients were diagnosed with FIGO 2018 stage ≥ 1B1, had visible tumor on MRI, and an imaging protocol comprising axial (oblique) T2-weighted imaging (T2WI) and diffusion-weighted imaging (DWI). (**b**) Within the study cohort, 92, 65 and 73 patients had available biomarker, mutational and transcriptomic data, respectively. Clinicopathological data including extensive follow up were available for all 132 included patients. In total, 18 patients had no biomarker, mutational nor transcriptomic data available. Cervical cancer depicted on magnetic resonance imaging (MRI) by sagittal T2-weighted imaging (T2WI) (**c**,**g**), axial oblique (with manually segmented tumor mask) T2WI (**d**,**h**), and axial oblique/axial diffusion weighted imaging (DWI) (**e**,**i**) with corresponding apparent diffusion coefficient (ADC) maps (**f**,**j**) in two different patients allocated to Cluster 1 and Cluster 3, respectively. The tumor masks were drawn from T2WI supported by high b-value and ADC map. (**c**–**f**) (Cluster 1 patient): A 38-year-old woman diagnosed with a FIGO stage IIB (tumor invading the parametrium) squamous cell carcinoma with MRI assessed maximum tumor size of 4.7 cm receiving radio-chemotherapy as primary treatment; the patient was alive without signs of recurrence 5 years after diagnosis. (**g**–**j**) (Cluster 3 patient): A 67-year-old woman diagnosed with FIGO stage IIA2 (tumor invading upper two thirds of the vagina and size > 4.0 cm) squamous cell carcinoma with MRI assessed maximum tumor size of 4.2 cm receiving radio-chemotherapy as primary treatment; the patient experienced recurrence and died from disease 88 days after primary diagnosis.

This retrospective study was performed under Institutional Review Board (IRB)-approved protocols (2015/2333 and 2018/591 Regional komité for forskningsetikk, Vest Norge (REK vest)) and in accordance with the Declaration of Helsinki. Written informed consent was obtained from all patients at primary diagnosis. All consenting patients admitted to Haukeland University Hospital between 2009 and 2017 with histologically confirmed cervical cancer and pretreatment pelvic MRI were initially enrolled (Fig. 1a, n = 437). All patients with (i) visible tumor (maximum tumor diameter range 8–207 mm) confirmed by two radiologists based on pelvic MRI including (ii) axial/axial oblique (relative to the long axis of the cervix) T2-weighted imaging (T2WI), and (iii) axial/axial oblique diffusion-weighted imaging (DWI) were included in the final study cohort (Fig. 1a, n = 132). As illustrated in Fig. 1b, mutational, transcriptomic and biomarker data were available for 65, 73 and 92 of these patients, respectively. As FIGO IA patients do not have visible tumors on MRI, the resulting study cohort consists of FIGO ≥ IB patients with higher age and more invasive primary treatment than the complete patient cohort (Supplementary Table 1). All patients were staged according to the FIGO 2018 criteria. Formalin fixed paraffin embedded (FFPE) tissue with corresponding HE-stained sections were collected from hospital archives for histopathological revision and tissue microarray (TMA) construction. An expert pathologist revised histological type and grade and assessed inflammatory reaction as previously described^3,14^. Disease-specific survival (DSS) was defined as time from primary treatment until death from cervical cancer or end of follow-up. Maximum tumor diameter was measured irrespective of plane on T2WI as previously reported^7^.

### Imaging protocol

Pretreatment pelvic MRI, performed as part of routine clinical workup, was acquired on scanners from different vendors (GE Healthcare, USA, RRID:SCR_000004; Siemens Healthineers, Germany; Philips Healthcare, Netherlands, RRID:SCR_008656), comprising 1.5 T (95/132 patients) or 3.0 T (37/132 patients) systems at three different hospitals in Western Norway. Imaging protocols and scanning parameters differed across scanners and hospitals. MRI included T2WI and DWI with two, three or four b-values (lowest b-value of 0 or 50; highest b-value of 800 or 1000). The *high b-value* dataset is defined as the DWI with the highest b-value for a particular patient. Vendor-provided software at the scanner was utilized to obtain apparent diffusion coefficient (ADC) maps from mono-exponential fits to the DWI data (for more details, see Hodneland et al.^15^).

### Tumor segmentation

The whole-volume of the cervical tumors was manually segmented on axial oblique (when available) or axial T2WI images (Fig. 1d and h), using the open-source software ITK-SNAP (v. 3.6.0; http://www.itksnap.org; RRID:SCR_002010). DWI series were available for visual inspection to verify tumor borders (Fig. 1e,f,I,j). The segmentations were performed by one of two experienced radiologists in 132 patients (K.W.L.: *n* = 67; N.L.: *n* = 65) with 12- and 7-years’ experience in pelvic MRI examination, respectively. The radiologists were blinded to clinicopathological patient information. The extracted 3D tumor mask was exported as a NifTI file (RRID:SCR_003141)^16^.

### Extraction of radiomic profiles

MR images were loaded from DICOM file format (https://dicomstandard.org; RRID:SCR_008925) using the open-source, Python-based package Imagedata (RRID:SCR_008394)^17^. An in-house developed Python script spatially aligned the high b-value DWI and ADC images with the grid of the axial/axial oblique T2WI by linear interpolation (RRID:SCR_008394), and radiomic feature values of these series were extracted from the resampled volumes. Radiomic features were extracted from the three imaging sequences (T2WI, high b-value DWI, and ADC) using the open-source software package Pyradiomics v3.0.1 (https://pyradiomics.readthedocs.io/en/latest)^18^. Default settings were used for all parameters except for sigma = [1,2,3] and binWidth = 10. Prior to radiomic feature extraction, each 3D image stack was divided by its own mean, then multiplied with a fixed factor of 100 to ensure a standardized value range in the radiomic feature extraction. The normalization aimed to reduce the influence of various field strengths, vendors, and acquisition protocols used in the MRI scans. Tumor shape features (*n* = 14 including tumor volume) were extracted from the T2WI only, while the remaining radiomic features were extracted for all three image sequences (*n* = 3 × 93), resulting in a total of 293 radiomic tumor features. The radiomic features are labelled according to their respective *feature group (glcm:* Gray Level Co-occurrence Matrix, *gldm*: Gray Level Dependence Matrix, *glrlm*: Gray Level Run Length Matrix, *glszm*: Gray Level Size Zone Matrix, *gtdm*: Neighboring Gray Tone Difference Matrix*)*, in agreement with naming conventions in Pyradiomics.

### Unsupervised clustering of radiomic features

Several phantom studies conducted under controlled environments have demonstrated that MR radiomic features are sensitive to variations in MR scanning protocol^19,20^. Also in our study, the algorithm tended to cluster patients based on MRI acquisition parameters, even after normalizing the image to its own average. To mitigate this undesirable effect, we assigned individual radiomic features as outcome variables in a linear regression model using the following explanatory parameters: MR voxel volume (slice thickness × pixel area), MR voxel anisotropy (slice thickness/pixel length), Repetition Time, Echo Time, Flip Angle, number of averages, field strength (1.5 T or 3.0 T), echo train length, and phase encoding direction. For DWI-derived features (high b-value features and ADC features) the highest b-value and the number of b-values were added to the list of explanatory variables. A fitted linear regression model provided a p-value suggesting whether the model had a significant explanatory effect. Adjusting for multiple testing using the false discovery rate (FDR)^21^ limited the normalization procedure to a list of radiomic features with statistically significant linear regression models. Assuming that data variation of scanner/protocol origin is incorporated within the linear model, we subtracted the linear prediction from the radiomic feature to obtain an unbiased radiomic feature estimate, independent of MRI acquisition parameters. Finally, we z-normalized each radiomic feature. Before normalization, 252/293 (86.0%) of the radiomic variables showed a significant linear relationship with MRI protocol parameters (multiple linear regression, FDR correction, α = 0.05). After normalization, none of the radiomic variables were associated with the MRI protocol parameters (multiple linear regression, FDR correction, α = 0.05). For reproducibility, the coefficients of the linear regression model are provided as Supplementary Table 2.

To minimize the amount of manually set parameters, we explored several automatic algorithms, i.e., the Calinski-Harabasz-, Davies-Bouldin-, and silhouette criterions as implemented in MATLAB (v R2022b; RRID:SCR_001622), to explore an optimal number of clusters (K) between 1 and 6. However, we encountered inconsistent results when using these three methods to determine the optimal number of clusters for our dataset. Additionally, even repeated analyses using the same method yielded different optimal K values. Due to these inconsistencies, we decided to opt for a user-defined value of K. With relatively few patients available for clustering, we aimed for the lowest possible K, thus maximizing the number of patients in each cluster. K = 2 yielded radiomic clusters with no difference in survival, whereas K = 3 yielded clusters exhibiting different patient outcomes, and patients were stratified into three radiomic clusters by K-medoids clustering (kmedoids in MATLAB) using a squared Euclidean distance measure and partitioning around medoids (PAM) to identify medoids^22^. Clusters were re-tested for association with the MRI acquisition parameters either by Chi-square (categorical variables) or by ANOVA1 test (continuous variables).

Cluster order was rearranged based on frequency of death-by-disease, leading to high-risk clusters associating with increasing cluster number (C1, C2, C3). A *feature ranking* (*FR*) array with 293 elements was associated with the Euclidean distance between cluster centroids $\mu \left({C}_{i}\right), i=\mathrm{1,2},3$, ${FR}_{k}\equiv \sqrt{{{\sum }_{i=1}^{3}\sum_{j=i+1}^{3}\left({\mu }_{k}\left({C}_{i}\right)-{\mu }_{k}\left({C}_{j}\right)\right)}^{2}}$. Higher values of *FR* indicate a larger separation of centroids between clusters, suggesting that a particular feature contributes significantly to the clustering. This assumption is legitimate only because of the previously acquired z-normalization. We sorted *FR* from high to low, representing more prominent features on the left side of the plots. Two intervals with particular contribution to the clustering were identified for thresholds *FR* > 1.85 (interval I) and 1.50 < *FR* < 1.85 (interval II). These intervals demonstrate the radiomic features that are most informative in distinguishing between patient clusters. Individual features were further labelled as shape features, or features uniquely originating from T2WI, high b-value DWI, and ADC maps.

### DNA and RNA isolation

An expert pathologist evaluated tumor cellularity on hematoxylin-stained sections from fresh frozen tumor biopsies. Total RNA and DNA were extracted from the fresh frozen tissue using the All-Prep DNA/RNA Mini Kit (Qiagen, Hilden, Germany) according to the manufacturer’s instructions. RNA quality was measured by Bioanalyzer 2100 (Agilent, Santa Clara, USA) and yield by Nanodrop 1000, (ThermoFisher Scientific, Waltham, USA) standards for the L1000 gene expression approach (Fig. 1b)^23^ (detailed L1000 protocol included in^24^).

### Whole exome sequencing

Whole exome sequencing (WES) requires high quality fresh frozen tissue with tumor purity above 50%. Of the 132 cervical tumors, 65 met this criterium and were subsequently selected for WES (Fig. 1b). The libraries were set up using KAPA Hyper Prep (100 ng input) and captured using the SeqCap EZ MedExome (Roche, Basel, Switzerland). The Illumina HiSeq 4000 (Twist: 100 × 2) was applied for sequencing. Bwa-mem (v.0.7.17) was used to align sequences to the human genome GRCh38, executed using the dockstore-cgpmap 3.3.0 pipeline. Raw read quality was assessed using the FASTQC and Picard software (v.2.17.0). The GATK (v4.2) was applied for depth and coverage analyses. MultiQC was used to generate data quality report and control factors such as duplicate and alignment rate. Total number of sequenced reads, unique reads, covered bases and coverage per base are summarized in Supplementary Table 3. Somatic variants were called using Mutect2 and Strelka. Mutect2 is part of the GATK docker container 4.2. Strelka (executed via 2.9.10 container from biocontainers.pro repository) was run using recommended default parameters for BWA aligned reads. Variant calling procedure included Panel of Normals generation, calculation of contamination tables and, finally, filtering using FilterMutectCalls function (GATK 4.2). In addition, the Mutect2 and Strelka variant overlap was limited to exome regions using MedExome hg38 bed files (Roche, Basel, Switzerland) and Bedtools intersect (v.2.27.1). Variants were annotated using Ensembl VEP following vcf2maf instructions (v.1.6.21).

### Transcriptomic analyses

In total, 73 of the 132 patients had available total RNA that met the quality (measured by Bioanalyzer 2100, RRID:SCR_019715, Agilent, Santa Clara, USA) and yield (measured by Nanodrop 1000, RRID:SCR_016517, ThermoFisher Scientific, Waltham, USA) standards for the L1000 gene expression approach (Fig. 1b)^23^ (detailed L1000 protocol included in:^24^). Differentially expressed genes were identified using the significance of microarrays (SAM) approach within the JExpress Software (http://www.molmine.com)^25^. Gene set enrichment analyses (GSEA) (JExpress Software) were scored by the Golub (signal-to-noise) methods and permuted on genes. The gene set collections C2 (curated gene sets), C5 (gene ontology gene sets), and Hallmarks (Molecular Signature database v4.0; RRID:SCR_016863) were queried for enriched gene sets^26^. The prognostic value of the 11 differentially expressed genes was assessed in an independent cervical cancer cohort published by the TCGA consortium^27^ within the Human Protein Atlas Database (https://www.proteinatlas.org/) (RRID:SCR_006710). An immune infiltration score was calculated for each patient with available L1000 data by using R (v3.6.3) (Massachusetts, USA) with the ESTIMATE (Estimation of Stromal and Immune cells in MAlignant tumor tissue using Expression) package ve1.0.13^28^.

### Immunohistochemistry for expression

Tissue microarrays (TMAs) were constructed, stained, and scored as previously described^14^. Successful TMA construction requires sufficient high-quality tissue and within the radiomic cohort, biomarker scores were available for 88, 92, 84 and 87 tumors for p53, PD-L1, HLA-DQB1 and LIMCH1, respectively^3,14^. The sections were scored according to the staining index (SI) combing staining intensity (0–3) and cell area (0 = no staining, 1 =  < 10%, 2 = 10–50%, 3 =  > 50%). For HLA-DQB1, a combined score of stroma and tumor staining intensity was applied. SI cut-off values defining high versus low protein expression for all antibodies are shown in Supplementary Table 4.

### Clinicopathological statistical analyses

Statistical data analyses were performed using the software package SPSS Statistics (Statistical Package of Social Science) v27.0 (IBM, Armonk, USA). All probability values were two-sided and considered statistically significant if < 0.05. Correlation between groups was assessed using Pearson’s χ^2^ or Fisher’s exact test, as appropriate for categorical variables. For continuous variables, the Mann–Whitney U or the Kruskal–Wallis test was applied, as appropriate. The Kaplan–Meier (product-limit) method was applied for patient survival analyses, and survival differences were calculated using the log-rank test (Mantel–Cox). Multivariate survival analyses were performed using the Cox proportional regression hazard ratio (HR) method, adjusting for FIGO stage and age at primary diagnosis.

## Results

### Unsupervised clustering of radiomic features identifies patient clusters with distinct risk profiles

**Figure 2:**
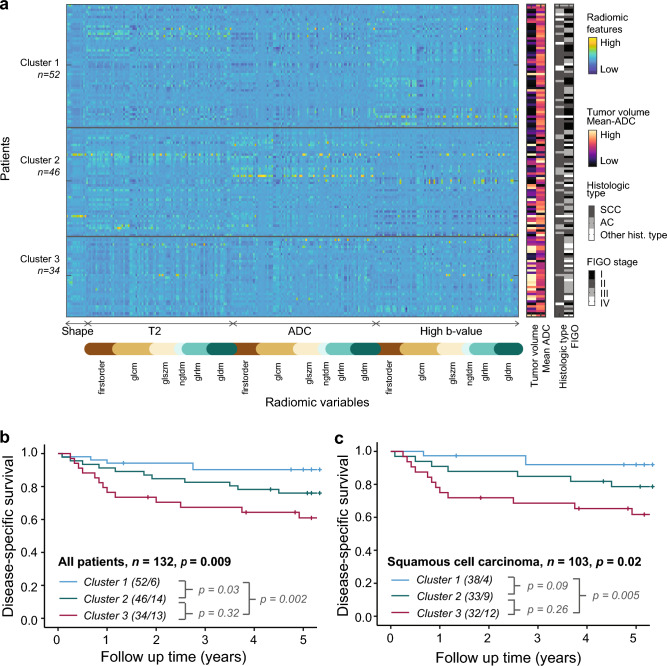
Unsupervised clustering of 293 radiomic features in 132 cervical cancer patients yields three distinct patient clusters exhibiting significant different risk profiles. (**a**) Unsupervised k-medoid clustering of 293 radiomic features identified three patient clusters exhibiting distinct radiomic profiles. Each vertical line (values along *x-axis*) represents one radiomic feature, and each horizontal line (values along *y-axis*) represents one patient. The tumor volumes, mean apparent diffusion coefficient (ADC) values, FIGO stage and histologic types for the same patients are displayed in right panels. The radiomic features extracted from T2-weighted images, apparent diffusion coefficient (ADC) maps and high b-value diffusion weighted images (DWI) are indicated by arrows. Similarly, radiomic feature groups (*firstorder, glcm, glszm, ngtdm, glrlm, gldm*) are shown by unique color codes. (**b**,**c**) Significantly different disease-specific survival (DSS) for patients within the three radiomic clusters was observed in the entire patient cohort (**b**) and for the subgroup of patients with squamous cell carcinoma (**c**). P-values of DSS differences between specific clusters are indicated in gray. Kaplan–Meier curves depict probability values from Mantel-Cox log-rank test comparing categories. Number of patients/events for each category is given in parentheses. ADC: apparent diffusion coefficient, FIGO: International Federation of Gynaecology and Obstetrics, SCC: squamous cell carcinoma, AC: adenocarcinoma, *glcm:* gray level co-occurrence matrix, *gldm*: gray level dependence matrix, *glrlm*: gray level run length matrix, *glszm*: gray level size zone matrix, *gtdm*: neighboring gray tone difference matrix.

Whole-volume MRI radiomic profiling was performed in 132 patients with visible cervical primary tumor at MRI. A total of 293 radiomic features were extracted from the segmented primary tumors (for details, see “Materials and methods”). Unsupervised clustering of the radiomic features yielded three distinct clusters (Clusters C1 [*n* = 52], C2 [*n* = 46] and C3 [*n* = 34]) exhibiting different radiomic tumor profiles (Fig. 2a). The clusters revealed no association with magnetic field strength or any of the other variables used in the normalization procedure by linear regression (FDR correction, chi-square test for categorical variables, ANOVA1 test for continuous variables, p > 0.052).

Patients in Cluster 2 and 3 had significantly poorer disease-specific survival than patients in Cluster 1, both when including all histologies (*n* = 132) (*p* = 0.009; Fig. 2b), and within the subgroup of squamous cell carcinomas (*n* = 103) (*p* = 0.02; Fig. 2c). Patients in Cluster 2/3 (combined) had an increased risk of death from cervical cancer with a hazard ratio (HR) of 3.33 (95% confidence interval [CI]: 1.37–8.07, *p* = 0.008; Table 1). Advanced FIGO 2018 stage (III/IV) and high age (years) also predict poor disease-specific survival with HRs of 4.52 (95% CI 2.04–10.03, p < 0.001) and 1.03 (95% CI 1.00–1.05, *p* = 0.02), respectively. In a multivariable analysis, including Cluster (2/3 vs 1), age (years) and FIGO 2018 stage (III/IV vs I/II), Cluster 2/3 independently predicts poor outcome (adjusted HR of 2.51, 95% CI 1.02–6.16; *p* = 0.045; Table 1).

**Table 1:** Cox regression analyses for prediction of disease-specific survival in relation to patient age, FIGO stage, radiomic cluster and histological subtype in cervical cancer patients (*n* = 132).

Variables	Unadjusted HR (95% CI)	P-value	Adjusted HR (95% CI)	P-value
Age at primary treatment (years)	1.03 (1.00–1.05)	**0.02**	1.03 (1.00–1.05)	**0.028**
FIGO 2018 stage I/II vs. III/IV (*n* = 71 vs 61)	4.52 (2.04–10.03)	** < 0.001**	3.75 (1.66–8.43)	**0.001**
Radiomic clusters 1 vs. 2/3 (*n* = 52 vs 80)	3.33 (1.37–8.07)	**0.008**	2.51 (1.02–6.16)	**0.045**
Histological type				
SCC (*n* = 103)	1	0.56		
AC (*n* = 21)	0.90 (0.35–2.36)	0.84		
Other (*n* = 8)	1.87 (0.57–6.21)	0.30		

FIGO: International Federation of Gynaecology and Obstetrics.

Significant values are in bold.

### Radiomic patient clusters exhibit distinct clinicopathological characteristics

The three clusters represent patient groups exhibiting distinct differences in age, FIGO 2018 stage, MRI-derived maximum tumor diameter and histological subtype (Table 2). Patients in Cluster 1 (*n* = 52) are characterized by low FIGO 2018 stage (stages I/II in 69% [36/52]), small tumor diameters (≤ 4 cm in 65% [34/52]) and rare histological types (in 12% [6/51]). Patients in Cluster 2 (*n* = 46) were younger (median age of 40 years vs. 47 and 54 years in Cluster 1 and 3, respectively) and more often diagnosed with adenocarcinomas (26% [12/43]). Cluster 3 patients (*n* = 34) were older (median age of 54 years) and more often presented with FIGO stage III/IV (in 68% [23/34]), tumors > 4 cm (in 88% [30/34]) and squamous cell carcinoma (in 94% [32/34]) (Table 2). Patients within the three clusters had similar body mass index (BMI) and histological grade. Median tumor volume is significantly higher in Cluster 2 and 3 than in Cluster 1 (Cluster 1: 6.8 ml, Cluster 2: 22.1 ml, Cluster 3: 58.8 ml; Kruskal–Wallis, p < 0.001; Supplementary Fig. 1a). Tumors in Cluster 3 have lower whole volume mean apparent diffusion coefficient (ADC) value (variable "firstorderMeanADC") compared to Cluster 1 tumors (*p* = 0.03, Supplementary Fig. 1b and Fig. 2a). Imaging findings in two different patients allocated to Cluster 1 and Cluster 3 are presented in Fig. 1c–j.

**Table 2:** Clinicopathological patient characteristics in the three radiomic clusters (using an unsupervised three-clustering approach).

Variables	Cluster 1 (n = 52) n (%)	Cluster 2 (n = 46) n (%)	Cluster 3 (n = 34) n (%)	P-value^a^
Median (range) age (years)	47 (29–95)	40 (23–85)	54 (28–85)	**0.002**^**b**^
BMI (kg/m^2^)				0.16
< 25	30 (58)	18 (39)	15 (44)	
≥ 25	22 (42)	28 (61)	19 (56)	
FIGO 2018 stage				**0.005**
IB	23 (44)	14 (30)	4 (12)	
II	13 (25)	10 (22)	7 (21)	
III	14 (27)	18 (39)	14 (41)	
IV	2 (4)	4 (9)	9 (26)	
Maximum tumor diameter (MRI)				** < 0.001**
≤ 4 cm	34 (65)	18 (39)	4 (12)	
> 4 cm	18 (35)	28 (61)	30 (88)	
Histologic type				**0.015**
Squamous cell carcinoma	38 (73)	33 (71)	32 (94)	
Adenocarcinoma	8 (15)	12 (26)	1 (3)	
Other histologic type	6 (12)	1 (2)	1 (3)	
Histologic grade				0.76
Grade 1/2	38 (79)	35 (83)	29 (85)	
Grade 3	10 (21)	7 (17)	5 (15)	
Primary treatment				0.004
Primary radiation ± chemotherapy	21 (40)	28 (61)	29 (85)	
Radical hysterectomy ± BSO/LA	26 (50)	15 (33)	3 (9)	
Simple hysterectomy ± BSO/LA	0 (0)	1 (2)	0 (0)	
Trachelectomy/conization ± LA	2 (4)	0 (0)	0 (0)	
Palliative treatment ± chemotherapy	3 (6)	2 (4)	2 (6)	

BSO: bilateral salpingo-oophorectomy, FIGO: International Federation of Gynaecology and Obstetrics, LA: lymphadenectomy.

Missing info: Grade, *n* = 9.

Significant values are in bold.

^a^Chi-square test.

^b^Kruskal–Wallis test.

### Radiomic features from T2WI are most important for the radiomic clustering

**Figure 3:**
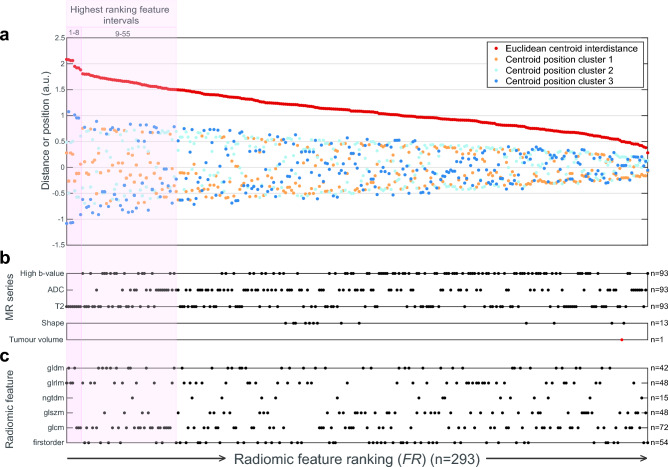
T2-derived radiomic features are most important for the radiomic clustering and extreme feature values associate with the high-risk cluster (Cluster 3). (**a**) Radiomic variables and their importance for the generated clusters, sorted according to descending feature ranking (*FR*) (from left to right). Higher Euclidean centroid distance (*red dots*) indicates larger separation of centroids and hence larger effect on the clustering. Orange, light blue and blue dots indicate centroid position of Cluster 1, 2 and 3, respectively, for each radiomic feature. Bar (**b**) indicates which MR series and (**c**) which feature group each radiomic feature is derived from. Two highest ranking feature intervals (shaded in pink) were recognized with characteristic profiles: (1) Features ranked from 1 to 8 have markedly elevated *FR*s and all features are derived from T2WI. (2) Features ranked 9–55, are also predominated by T2WI. (3) In both intervals, Cluster 3 (blue dots) associates with extreme (both positive and negative) centroid values. The number of features (n) derived from the respective MR series (**b**) and radiomic feature groups (**c**) is given in the plot. Tumor volume (plotted value highlighted in red) was ranked as 280 (out of the 293 features) for feature importance for the derived clustering (**b**). A detailed description of all 293 radiomic features and their contribution to the radiomic clustering is presented in Supplementary Note 1 and Supplementary Fig. 2, respectively. *FR*: feature ranking, glcm*:* gray level co-occurrence matrix, *gldm*: gray level dependence matrix, *glrlm*: gray level run length matrix, *glszm*: gray level size zone matrix, *ngtdm*: neighboring gray tone difference matrix.

The mean value (i.e., cluster centroid) for each radiomic feature within the clusters (C1, C2 & C3), ordered based on declining feature ranking (*FR*), is shown in Fig. 3 (for details, see “Materials and methods”). Eight single radiomic features (interval I, consecutively ranked from 1–8) with markedly elevated *FR*s (Fig. 3a, ranking 1–8) are all derived from the T2WI (Fig. 3b and Supplementary Table 5a and b). Among the next 47 radiomic features (interval II, consecutively ranking 9–55), T2WI features were also predominant (Fig. 3b, Supplementary Table 5a and b). No statistical difference in ranking across feature groups was found (Fig. 3c, Supplementary Table 5a). Extreme centroid values, either positive or negative, occurred most frequently in the high-risk Cluster 3 and least frequently in the low-risk Cluster 1 (Fig. 3a, Supplementary Table 6). A total of 189/293 (64.5%) radiomic features were statistically associated with tumor volume (Spearman correlation, FDR correction, α = 0.05). Features statistically associated with tumor volume did not have higher *FR* values in terms of ranking compared to those that were not associated with tumor volume (Kruskal–Wallis, *p* = 0.37). Furthermore, tumor volume had low feature ranking (280 out of 293) (Fig. 3b; Supplementary Table 5b), and the three clusters had similar mean tumor volumes (ANOVA test for differences in means *p* = 0.13; Supplementary Table 5b), all supporting that the clustering was not primarily driven by differences in mean tumor volume.

### Mutational analyses comparing the radiomic patient clusters reveal druggable targets

**Figure 4:**
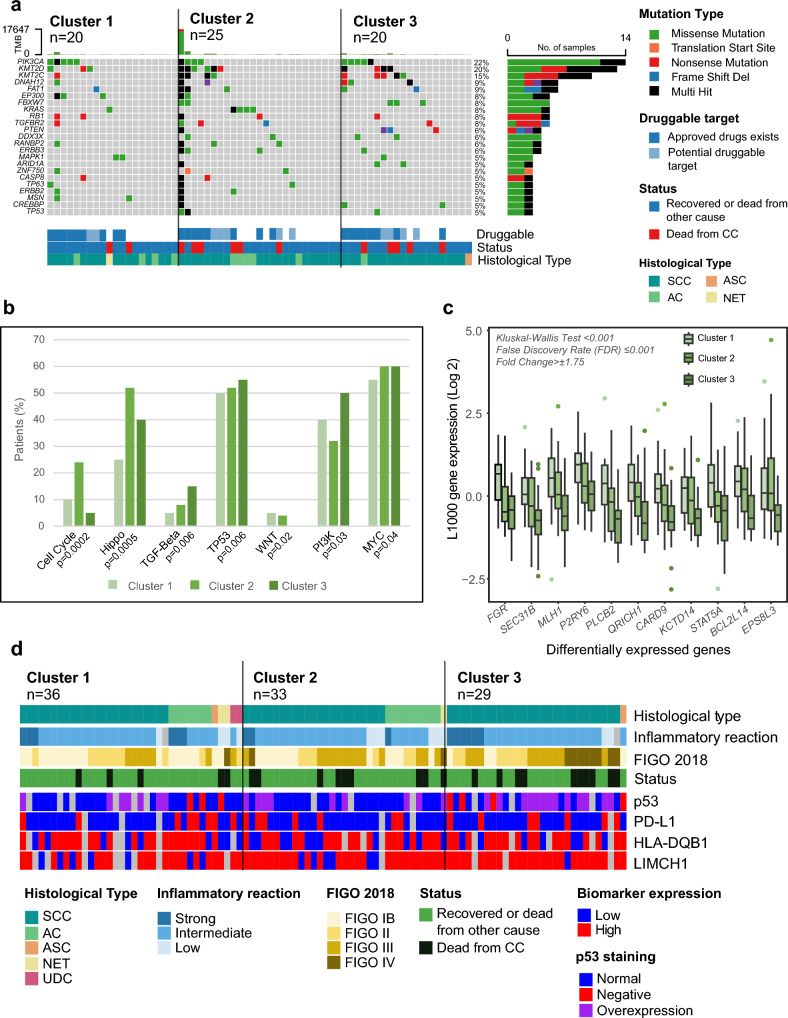
Genomic, transcriptional, and molecular characterization of clusters reveal immunotherapy, CDK4/6 and YAP-TEAD inhibitors and p53 pathway plausible treatment strategies within clusters. (**a**) Oncoplot displaying the top 20 most frequently significantly mutated cervical cancer genes in relation to radiomic clusters. 16 of these genes (*) contain mutations that are druggable as defined by the Human Protein Atlas (https://www.proteinatlas.org/) (for more details, see Supplementary Table 7). (**b**) Proportion of patients with mutations within main oncogenic pathways in relation to radiomic cluster. (**c**) Distribution of L1000 log 2 expression levels of the 11 differentially expressed genes ((False Discovery Rate (FDR) < 0.01, Fold Change >  ± 1.75) relative to radiomic cluster. The distribution of gene expression values is significantly different between radiomic clusters (Kruskal–Wallis test; p ≤ 0.001). (**d**) Heatmap of protein expression for known prognostic biomarkers (p53, PD-L1, HLA-DQB1, LIMCH1) in cervical cancer cases ordered by, radiomic cluster, histological type, inflammatory reaction and FIGO2018 stage. Grey boxes indicate no biomarker data available. p53 aberrations (negative or strong staining) are more common in Cluster 3 tumors in 68% (17/27) vs. in 30% (18/61) of Cluster 1/2 tumors (^γ2^ test; *p* = 0.004). High LIMCH1 protein expression tended to be more common in Cluster 3 tumors (in 96% (25/26) vs. in 79% (48/61) of Cluster 1/2 tumors) (Fisher’s Exact Test; *p* = 0.056). AC: adenocarcinoma; ASC: adenosquamous carcinoma; CC: cervical cancer; NET: neuroendocrine tumor; SCC: squamous cell carcinoma; UDC: undifferentiated carcinoma.

To gain insight into the genomic landscape and reveal potential targets for treatment in the different clusters, mutations in known significantly mutated genes (SMGs) in CC^29^ were compared between clusters (Fig. 4a). Cluster 1 tumors had the lowest mutational frequencies within these genes as compared to Cluster 2 and 3 tumors. In a broader analysis including mutational data for all genes, *FIZ1* and *ZNF275* had significantly higher- and *RYR1* lower mutational frequency in Cluster 1 compared to those in Cluster 2 and 3 tumors (Supplementary Fig. 2). The burden of significantly mutated cervical cancer genes including *KMT2D* and *KRAS* was highest in Cluster 2. Interestingly, five of six patients in Cluster 2 who died from disease, had mutations in druggable targets (Fig. 4a, Supplementary Table 7). Cluster 2 also contained one ultra-mutated tumor with 17,647 non-silent mutations. In total, 32 genes with significantly higher mutation frequencies were detected in Cluster 2 tumors (Supplementary Fig. 2). Among Cluster 3 tumors, druggable alterations (e.g., *PIK3CA*, *ERBB2*, *DDX3X* and *CREBBP* mutations) were detected in three of five patients who died from CC. The mutational frequency of *NEDD4L*, *B3GNT8*, *CDHR5*, *CNGC3* and *NDUFS2* was significantly higher in Cluster 3 tumors than in Cluster 1 and 2 tumors. A broader analysis of oncogenic pathway alterations according to clusters revealed that the distribution of cell cycle pathway mutations was significantly associated with radiomic clusters (Fig. 4b). Specifically, Cluster 2 had higher levels of cell cycle and Hippo pathway mutations, whereas Cluster 3 had higher levels of TGF-beta and PI3K pathway mutations. In Cluster 1, a low or intermediate number of oncogenic pathway mutations were detected.

### Transcriptomic analyses reveal distinct pathway activation within clusters

One gene was significantly differentially expressed (*EPS8L3*) between Cluster 2 and 3 and ten genes (FGR*, SEC31B*, MLH1*, *P2RY6*, PLCB2*, *QRICH1*, *CARD9*, *KCTD14*, STAT5A* and BCL2L14*) between Cluster 1 and 3 (FDR < 0.01, Fold Change >  ± 1.75, Fig. 4c), six of which (*) associated with better survival in the TCGA cervical cancer cohort (*n* = 304, *p* < 0.02, https://www.proteinatlas.org/). Gene set enrichment analyses (GSEA), revealed that Cluster 1 tumors associate with immune cell (e.g. interferon gamma signaling, antigen presentation, granules, leukocytes, adaptive immune response) and granule membrane activity (Supplementary Tables 8/9a and b), Cluster 2 tumors associate with DNA replication, protein translation, membrane transport and cell cycle (Supplementary Tables 8/9a and c), and Cluster 3 tumors with metabolism, squamous histology, translation and hypoxia (Supplementary Tables 8/9b and c). Overall, these gene expression analyses indicate that Cluster 1 tumors have higher immune activation and that Cluster 2 and 3 tumors express genes related to increased proliferation and metabolism.

### The high-risk radiomic Cluster 3 associates with abnormal p53 and high LIMCH1 protein expression

To further characterize the distinct clusters, the expression of known molecular biomarkers in CC^3,14^, that is, p53, PD-L1, HLA-DQB1, and LIMCH1, was investigated (Fig. 4d and Supplementary Table 4). p53 overexpressing tumors indicate *TP53* mutation and p53 negative tumors indicate *TP53* deletion^3,30^. For Cluster 1 and 2 tumors, p53 was overexpressed or negative in 26% (16/61) and 5% (3/61), respectively, whereas for Cluster 3 tumors, 63% (17/27) were overexpressed (13/29) or negative (4/29). The immune activation markers HLA-DQB1 and the immune checkpoint inhibitor response marker PD-L1 were evenly distributed between clusters; however, the marker LIMCH1, indicating poor prognosis, was highly expressed in Cluster 3 tumors (96%; (25/26) compared with Cluster 1 (76%: 22/29) and Cluster 2 (81%, 26/32) tumors (*p* = 0.06, Supplementary Table 4).

## Discussion

This study demonstrates that whole-volume magnetic resonance imaging (MRI) radiomic tumor profiling captures microstructural tumor features that are closely linked to clinical characteristics and outcomes in patients with uterine cervical cancer. Based on radiomic features only, unsupervised clustering yielded three distinct patient groups exhibiting highly different clinicopathological characteristics and phenotypes, which were also reflected in survival. Importantly, the same radiomic clusters associated with specific genomic alterations and transcriptional programs. This suggests that the radiogenomic approach presented herein may be used to non-invasively identify specific cell signaling profiles that can be targeted by novel treatments in cervical cancer.

Several studies have reported a potential value of MRI based radiomic tumor profiling for assessing therapeutic response^31,32^ and for predicting survival^33–35^ and recurrence/metastatic spread^36–38^ in cervical cancer. Unfortunately, poor reproducibility is a major general challenge with radiomics, which may be due to variability in image acquisitions, scanner model/manufacturer and protocol settings^39,40^, image analyses (e.g. manual *versus* machine learning based tumor segmentations^41^) and the statistical modelling employed^42,43^. Hence, an important step when assessing the value of radiomics in cervical cancer is to develop and employ robust and standardized methodologies to increase the reproducibility and transferability of findings to independent patient cohorts. Therefore, in this study, we extracted and reported radiomic features following the recommendations by the Image Biomarker Standardization Initiative (IBSI; https://theibsi.github.io/). We also made extensive efforts to reduce protocol/scanner-induced bias by fitting a linear model to each of the radiomic features using scanner protocol as predictive variables. Subtracting this model from the radiomic feature values yielded a linearly unbiased data estimate leading to clusters that were not associated with MRI protocol. By using this approach, the observed cluster distribution is more likely to capture radiomic features reflecting biologic variation rather than scanner-variations.

Large MRI-measured tumor size is known to predict high-risk cervical cancer disease^5,8^. Interestingly, we found no overrepresentation of tumor-volume-associated radiomic features among the high-ranked cluster-driving features. We have also demonstrated that clustering based on the full radiomic profile provides clusters with substantially different composition than clustering based on tumor volume alone. This suggests that volume-independent radiomic features may capture microstructural tumor characteristics relevant to the clinical phenotype. Furthermore, we found that within the top-ranked cluster-driving features, the absolute feature values in Cluster 1 were lower than in Cluster 2 and Cluster 3. Interestingly, this suggests that at least for the top-ranked radiomic features, high-risk patients presented more extreme values than low-risk patients.

Both T2-weighted imaging (T2WI) and DWI are recommended MRI sequences used for initial cervical cancer staging^6^, with T2WI considered the mainstay for detecting and assessing the extent of cervical tumors. Interestingly, T2WI predominated among the top-ranked cluster-driving features in this study. This may be due to T2WI yielding superior soft-tissue resolution, that may be important for the retrieval of radiomic profiles. In comparison, DWI yields lower soft-tissue resolution with less detailed anatomic information. Thus, in spite of DWI putatively being closely linked to tumor microstructure, -cellularity and -cellular membrane integrity by its quantification of water mobility within the tumor^44^, the DWI radiomic profiles may be less able to capture phenotypic information.

Unsupervised clustering of 293 radiomic features in 132 cervical carcinomas yielded three distinct radiomic clusters comprising patients with significantly different survival rates. Cluster 1 patients were characterized by favorable survival and had small tumors with enriched immune cell signaling. Consistent with this, immune signaling has previously been linked to favorable survival in cervical cancer^14,45^. Cluster 2 patients were characterized by intermediate-risk, low age, and adenocarcinoma histology. Transcriptional analyses revealed upregulated proliferation- and cell cycle signaling, and genomic analyses identified accumulation of cell cycle- and Hippo pathway mutations. Aberrant Hippo signaling may lead to hyperproliferation, cellular invasion, metastasis, and chemotherapeutic resistance^46^. Taken together, Cluster 2 tumors appear to be characterized by aberrant cell cycle regulation which leads to high proliferation enabling cellular invasion and metastasis. This together with chemotherapy resistance could partly explain the poorer survival rate in this group. Furthermore, this finding suggests that switching from chemotherapy-based treatments to cell cycle targeting compounds (e.g., CDK4/6 inhibitors^47^) and Hippo pathways (e.g., YAP-TEAD inhibitors^46^) could prove beneficial in several Cluster 2 patients.

Cluster 3 patients were characterized by high-risk features including older age, advanced stage, and large tumor size. Surprisingly, a higher proportion of squamous cell carcinomas (SCC) was seen in Cluster 3, even though the SCC subtype allegedly indicates a more favorable prognosis. However, a significant proportion of SCC patients in Cluster 3 (12/32: 38%) died from CC. Thus, radiomic profiling may aid in predicting poor outcomes in patients with SCC. Our genomic and transcriptomic analyses indicate that Cluster 3 tumors exhibit aberrant p53-, MYC- and MTORC1 signaling caused by mutations, some of which may be responsible for the aggressive phenotype. Aberrant p53 signaling was confirmed by IHC where we found aberrant p53 expression (negative or overexpressed protein level) in 68% of Cluster 3 tumors as opposed to only 30% in Cluster 1/2 tumors combined. Whether effective drugs that target these aberrations could be developed for Cluster 3 tumors, warrants further investigation.

This study has some limitations. For radiomic profiling we used tumor masks drawn on T2WI only; these masks were placed on interpolated DWI resampled to the same slice thickness and voxel size as those of the corresponding T2WI. This process of pixel resampling and interpolation may have slightly influenced the accuracy of the tumor masks on the DWI and the corresponding DWI radiomic profiles. However, manual tumor segmentations of all series would be highly time consuming and was thus considered unfeasible. Furthermore, only half of the included patients had overlapping genomic- and transcriptomic- data, thus hampering the genomic profiling of the clusters. Currently, such genomic profiling requires high quality fresh tissue which is challenging to obtain in retrospective studies. Future improvements in tissue sampling and DNA/RNA extraction protocols will enable comprehensive genomic profiling in prospective cancer cohorts. Finally, ideally the described MRI radiomic model needs to be validated in independent and prospective cervical cancer patient series, and this is planned in future follow-up studies.

In conclusion, this study shows how non-invasive preoperative MRI radiomic profiling yields distinct radiomic patient clusters with different risk-profiles and prevalence of molecular- and genetic tumor aberrations in cervical cancer. Altogether, this information may inform the selection of patients for more individualized and targeted treatment schemes (e.g., tailored surgery, radio-chemotherapy, immunotherapy, CDK4/6 and YAP-TEAD inhibitors, and p53 pathway-targeting treatments). However, the described MRI radiomic prognostic and predictive model needs to be validated in independent cervical cancer patient series to prove its role as a clinically useful tool.

### Supplementary Information

Supplementary Information.

## Data Availability

The datasets generated during and/or analyzed during the current study are provided within the supplementary information files or from the corresponding author on reasonable request.
