# Supplementary material for: Radiomic profiles improve prognostication and reveal targets for therapy in cervical cancer
Source: Sci Rep. 2024 May 17;14:11339. doi: 10.1038/s41598-024-61271-4 (PMC11101482; doi:10.1038/s41598-024-61271-4)
Supplement: Supplementary file 1 — Supplementary Information. [file 41598_2024_61271_MOESM1_ESM.pdf]

Supplementary Material

Radiomic profiles improve prognostication and reveal targets for therapy in cervical cancer

Mari K. Halle[1,2], Erlend Hodneland[3,4], Kari S. Wagner-Larsen[3,5], Njål G. Lura[3,5], Kristine E. Fasmer[3,5], Hege F. Berg[1,2], Tomasz Stokowy[6,7], Aashish Srivastava[6,7], David Forsse[1,2], Erling A. Hoivik[1,2], Kathrine Woie[2], Bjørn I. Bertelsen[8], Camilla Krakstad[1,2], and Ingfrid S. Haldorsen[3,5]

[1]Centre for Cancer Biomarkers, Department of Clinical Science, University of Bergen, Bergen, Norway.
[2]Department of Obstetrics and Gynecology, Haukeland University Hospital, Bergen, Norway.
[3]Mohn Medical Imaging and Visualization Centre, Department of Radiology, Haukeland University Hospital, Bergen, Norway.
[4]Department of Mathematics, University of Bergen, Bergen, Norway
[5]Section of Radiology, Department of Clinical Medicine, University of Bergen, Bergen, Norway
[6]Genomics Core Facility, Department of Clinical Science, University of Bergen, Bergen, Norway
[7]Section of Bioinformatics, Clinical Laboratory, Haukeland University Hospital, Bergen, Norway
[8]Department of Pathology, Haukeland University Hospital, 5021 Bergen, Norway

Table of content

Supplementary Figure 1 …………………….…………………….………….. 2
Supplementary Figure 2 …………………….…….……………….………….. 3
Supplementary Table 1 ..……………..………………………………….…... 4
Supplementary Table 2 ..………….………………………………………… 5-20
Supplementary Table 3 ....……………………………………………….... 21-25
Supplementary Table 4 ..…………………………………………………… 26
Supplementary Table 5 ……………………………………………….…..... 27-31
Supplementary Table 6 ..………………..…………….…..………………… 32
Supplementary Table 7 ..………………………….…...……………………. 33
Supplementary Table 8 ..……………..………………...…………………. 34-36
Supplementary Table 9 ……………..………………...………………….. 37-38
Supplementary Note ……………………..……………………………… 39-41

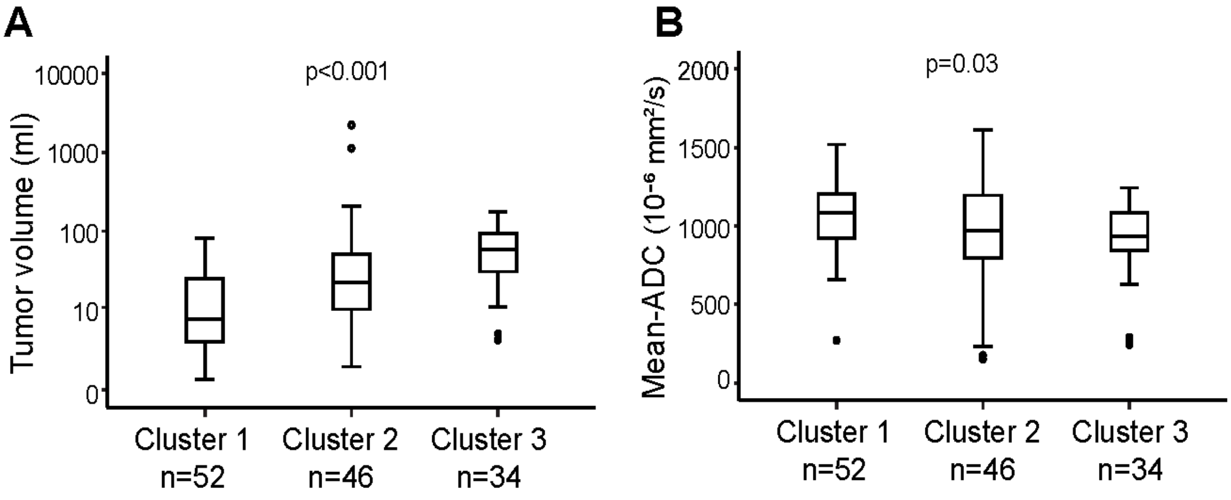

Supplementary Figure 1: Large tumour volume and low mean-ADC characterize the high-risk clusters. Distribution of tumour volume (**A**, variable "TumorVolume") and whole-volume ADC-values (**B**, variable "firstorderMeanADC") for the three radiomic cluster. Tumour volume is presented on a logarithmic scale. Central line in boxes is the median. All p-value are based on Independent—Samples Kruskal-Wallis Tests. Abbreviations: ADC: Apparent Diffusion Coefficient.

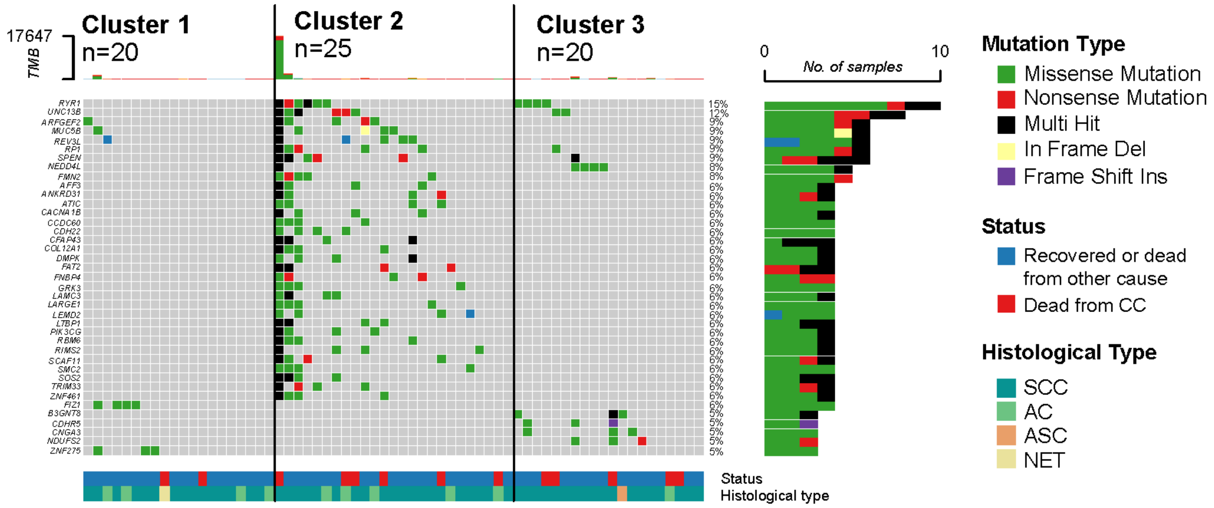

Supplementary Figure 2. Differentially mutated genes according to radiomic clusters. Oncoplot displaying genes with significantly differential mutational frequency between clusters (Fisher's Exact Test, p<0.01). Patients are ordered based on radiomic cluster and frequency of mutations. Histological type and status are displayed on the bottom. Abbreviations: SCC: Squamous cell carcinoma, AC: Adenocarcinoma, ASC: Adenosquamous carcinoma, NET: Neuroendocrine tumour.

Supplementary Table 1. Clinicopathological characteristics for all cervical cancer patients (n=437) treated at the hospital (whole cohort) from 2009 to end of 2017 and for the subgroup comprising the radiomics cohort (n=132).

**Variables**	**Whole cohort (n=437) n (%)**	**Radiomics cohort (n=132) n (%)**	***P-value*** [a]
**Median (range) age (yrs.)**	43 (23-95)	48 (23-95)	**0.03** [b]
**FIGO (2018) stage**			**<0.001**
IA	117 (27)		
IB	149 (34)	41 (31)	
II	53 (12)	30 (23)	
III	85 (19)	46 (35)	
VI	33 (8)	15 (11)	
**Primary Treatment**			**<0.001**
Primary radiation ± chemotherapy	146 (33)	78 (59)	
Radical hysterectomy ± BSO/LA	186 (43)	44 (33)	
Simple hysterectomy ± BSO/LA	48 (11)	0 (0)	
Trachelectomy/Conization ± LA	42 (10)	3 (1)	
Palliative treatment ± chemotherapy	15 (3)	7 (5)	
**Histological type**			0.68
Squamous cell carcinoma	330 (76)	103 (78)	
Adenocarcinoma	84 (19)	21 (16)	
Other histologic type	23 (5)	8 (6)	
**Histological grade**			0.57
Grade 1/2	308 (84)	102 (82)	
Grade 3	57 (16)	22 (18)	

a: Chi-square test

b: Unpaired t-test

Abbreviations: FIGO: International Federation of Gynecology and Obstetrics, BSO: Bilateral salpingo-oophorectomy, LA: Lymphadenectomy

Missing info in whole cohort: Grade, n=72

Missing info in radiomic cohort: Grade, n=8

Supplementary Table 2. Coefficients of the linear regression model used for normalization of the radiomics data. 86% of the radiomic features were statistically associated with MRI scanning protocol (after false discovery rate correction).

Row	(Intercept)	TR	TE	FA	NumberOfAverages	EchoTrainLength	BvalHigh	nBvals	VoxelVolume	Anisotropy	PhaseEncodingDirection_ROW	FieldStrength_3	p-value
firstorder10PercentileADC	422	-0.01	1.44	-0.75	-7.68	-0.36	-0.12	-22.40	-1.17	-18.03	-2.74	-6.51	0.00
firstorder10PercentileDWI	175	-0.02	-0.12	0.55	-1.20	0.08	-0.04	-2.09	1.54	11.37	-11.77	-15.90	0.00
firstorder10PercentileT2	58.33	0.00	0.41	-0.06	-0.61	-0.82	0.00	0.00	1.60	-0.15	-10.44	-0.30	0.16
firstorder90PercentileADC	807	-0.02	3.05	-1.48	-15.45	-0.76	-0.22	-33.50	-3.42	-45.11	13.09	5.57	0.00
firstorder90PercentileDWI	-461.11	0.00	0.41	1.39	4.49	0.58	0.44	-21.75	4.89	76.58	21.10	92.18	0.00
firstorder90PercentileT2	120	0.00	0.76	-0.19	0.73	-1.82	0.00	0.00	-0.26	-0.16	-26.27	0.09	0.00
firstorderEnergyADC	1.22E+10	-4.48E+05	7.72E+07	-4.44E+07	-5.79E+08	-1.86E+07	-6.29E+05	-1.01E+09	-2.98E+07	-7.04E+08	3.30E+08	-2.24E+08	0.01
firstorderEnergyDWI	-1.93E+10	-8.18E+05	2.35E+08	-5.28E+06	-1.01E+09	-6.26E+07	7.35E+06	-2.57E+08	1.59E+08	3.90E+09	4.49E+08	1.84E+09	0.00
firstorderEnergyT2	-7.08E+09	4.60E+04	4.52E+07	-1.55E+07	-9.29E+07	-4.83E+07	0.00E+00	0.00E+00	1.46E+09	8.12E+08	-1.19E+09	1.32E+09	0.00
firstorderEntropyADC	6.56	0.00	0.03	-0.01	-0.11	0.00	0.00	-0.11	-0.03	-0.39	0.22	0.27	0.00
firstorderEntropyDWI	0.94	0.00	0.00	0.00	0.00	0.00	0.00	-0.06	0.03	0.25	-0.01	0.68	0.00
firstorderEntropyT2	3.34	0.00	0.01	0.00	0.02	-0.02	0.00	0.00	-0.04	-0.02	-0.41	-0.02	0.00
firstorderInterquartileRangeADC	132	0.00	0.75	-0.27	-2.98	-0.12	-0.02	-4.86	-1.16	-10.04	5.12	4.00	0.01
firstorderInterquartileRangeDWI	-519.08	0.01	0.29	0.73	6.97	0.37	0.36	-8.88	1.14	45.00	21.05	53.90	0.00

firstorderInterquartileRangeT2	29.26	0.00	0.21	-0.07	0.47	-0.51	0.00	0.00	-0.55	0.25	-8.41	0.66	0.00
firstorderKurtosisADC	15.34	0.00	0.02	-0.03	-0.20	-0.02	-0.01	-0.22	0.04	-0.39	0.35	0.95	0.07
firstorderKurtosisDWI	5.10	0.00	0.00	0.00	-0.04	0.00	0.00	-0.07	0.00	-0.07	0.92	0.10	0.09
firstorderKurtosisT2	-3.18	0.00	-0.05	0.01	0.46	0.14	0.00	0.00	0.31	0.34	0.73	0.22	0.02
firstorderMaximumADC	1638	-0.04	5.56	-3.72	-34.65	-1.98	-0.49	-61.14	-4.90	-89.78	38.66	69.24	0.00
firstorderMaximumDWI	-448.27	-0.01	1.40	1.09	-5.15	1.13	0.55	-30.18	8.98	84.18	100	291	0.00
firstorderMaximumT2	157	0.02	0.40	-0.44	7.05	-0.87	0.00	0.00	12.30	5.76	-57.13	19.63	0.00
firstorderMeanAbsoluteDeviationADC	116	0.00	0.52	-0.24	-2.39	-0.13	-0.03	-3.53	-0.71	-8.14	4.74	4.43	0.00
firstorderMeanAbsoluteDeviationDWI	-213.63	0.01	0.16	0.28	2.28	0.17	0.16	-5.85	0.95	21.30	10.95	35.02	0.00
firstorderMeanAbsoluteDeviationT2	17.93	0.00	0.10	-0.04	0.53	-0.28	0.00	0.00	-0.53	0.06	-4.87	0.16	0.00
firstorderMeanADC	585	-0.02	2.09	-1.01	-11.02	-0.47	-0.16	-27.40	-2.17	-30.06	4.24	-4.89	0.00
firstorderMeanDWI	-170.12	-0.01	0.36	1.07	1.87	0.33	0.20	-13.23	2.98	47.44	2.28	27.72	0.00
firstorderMeanT2	84.38	0.00	0.59	-0.12	-0.11	-1.27	0.00	0.00	1.53	0.02	-18.77	0.56	0.01
firstorderMedianADC	562	-0.02	1.93	-0.92	-10.67	-0.39	-0.15	-26.40	-2.04	-29.36	3.52	-9.03	0.00
firstorderMedianDWI	-230.15	-0.01	0.59	1.25	2.95	0.31	0.22	-15.26	2.50	53.21	-1.52	12.58	0.00
firstorderMedianT2	82.04	0.00	0.60	-0.12	-0.49	-1.24	0.00	0.00	2.35	0.06	-19.30	1.07	0.01
firstorderMinimumADC	78.36	0.00	-0.49	0.32	3.42	0.15	-0.04	-10.28	0.03	9.61	-14.69	-31.57	0.00
firstorderMinimumDWI	149	-0.01	0.52	0.21	-1.27	-0.07	-0.08	-0.18	0.24	-3.24	-11.79	-22.18	0.06
firstorderMinimumT2	-40.45	0.00	0.34	0.08	-0.50	-0.26	0.00	0.00	-0.51	1.01	2.83	-0.90	0.16

firstorderRangeADC	1559	-0.04	6.06	-4.03	-38.07	-2.13	-0.45	-50.85	-4.93	-99.39	53.34	101	0.00
firstorderRangeDWI	-596.90	0.00	0.89	0.89	-3.88	1.20	0.63	-30.00	8.74	87.42	112	313	0.00
firstorderRangeT2	198	0.02	0.06	-0.52	7.55	-0.61	0.00	0.00	12.81	4.75	-59.96	20.53	0.00
firstorderRobustMeanAbsoluteDeviationADC	61.01	0.00	0.33	-0.13	-1.32	-0.06	-0.01	-2.12	-0.49	-4.59	2.26	2.08	0.00
firstorderRobustMeanAbsoluteDeviationDWI	-196.14	0.00	0.09	0.28	2.65	0.14	0.14	-3.90	0.51	18.12	7.96	23.50	0.00
firstorderRobustMeanAbsoluteDeviationT2	13.04	0.00	0.09	-0.03	0.20	-0.23	0.00	0.00	-0.26	0.09	-3.46	0.29	0.00
firstorderRootMeanSquaredADC	613	-0.02	2.20	-1.07	-11.61	-0.51	-0.16	-28.04	-2.34	-32.11	6.20	-3.26	0.00
firstorderRootMeanSquaredDWI	-228.27	-0.01	0.39	1.10	2.48	0.38	0.24	-15.78	3.33	53.68	7.45	42.17	0.00
firstorderRootMeanSquaredT2	87.40	0.00	0.59	-0.13	0.15	-1.31	0.00	0.00	1.48	0.07	-19.73	0.59	0.00
firstorderSkewnessADC	4.89	0.00	0.00	-0.01	-0.03	-0.01	0.00	-0.08	0.02	0.04	0.01	0.58	0.02
firstorderSkewnessDWI	0.48	0.00	0.01	-0.01	-0.05	0.00	0.00	-0.02	0.00	-0.18	0.33	0.39	0.03
firstorderSkewnessT2	-1.23	0.00	-0.01	0.00	0.13	0.02	0.00	0.00	-0.15	0.04	0.26	0.01	0.01
firstorderTotalEnergyADC	1.E+11	3.E+06	2.E+09	-4.E+08	-1.E+10	-2.E+08	-6.E+07	-2.E+10	2.E+09	-8.E+09	2.E+09	-2.E+10	0.02
firstorderTotalEnergyDWI	-2.E+11	2.E+07	4.E+09	1.E+09	-2.E+10	-9.E+08	-3.E+08	-2.E+10	1.E+10	8.E+10	3.E+10	-9.E+10	0.00
firstorderTotalEnergyT2	-6.E+09	18519	19090827	-10410332	-1299128	-53750628	0	0	2.E+09	7.E+08	-9.E+08	1.E+09	0.00
firstorderUniformityADC	0.04	0.00	0.00	0.00	0.01	0.00	0.00	0.00	0.00	0.03	-0.02	-0.02	0.00
firstorderUniformityDWI	0.18	0.00	0.00	0.00	0.00	0.00	0.00	0.00	0.00	-0.01	0.01	-0.02	0.00
firstorderUniformityT2	0.10	0.00	0.00	0.00	0.00	0.00	0.00	0.00	0.01	0.00	0.04	0.00	0.00
firstorderVarianceADC	21992	-0.67	71.17	-51.87	-512.61	-30.28	-5.69	-979.39	-101.49	-140	860	298	0.06

										9.20			
firstorderVarianceDWI	-85998	3.19	184.11	90.37	742.41	16.71	45.52	-3244.12	112	7258	6011	8360	0.00
firstorderVarianceT2	413	0.07	4.29	-2.27	62.29	-13.16	0.00	0.00	-30.80	16.14	-330.62	26.91	0.00
glcmAutocorrelationADC	1654	-0.05	7.36	-4.78	-48.37	-2.32	-0.36	-83.79	-7.08	-119.03	57.41	40.11	0.00
glcmAutocorrelationDWI	-3496	0.05	6.94	5.72	20.22	-0.20	1.97	-126.98	9.58	372	202	280	0.00
glcmAutocorrelationT2	148	-0.01	0.56	-0.38	0.60	-1.89	0.00	0.00	7.96	0.56	-42.70	7.12	0.00
glcmClusterProminenceADC	2.15E+06	-7.36E+01	4.72E+03	-7.01E+03	-7.66E+04	-2.30E+03	157	-2.04E+05	-4.31E+03	-1.31E+05	4.80E+04	-8.96E+04	0.87
glcmClusterProminenceDWI	-2.25E+07	1.13E+03	1.07E+05	1.36E+04	2.24E+04	-1.26E+04	1.02E+04	-1.14E+06	-2.36E+04	1.34E+06	1.83E+06	1.11E+05	0.00
glcmClusterProminenceT2	-2744	0.83	30.23	-35.17	290.31	-1.90	0.00	0.00	409	526	-2467.31	1041	0.01
glcmClusterShadeADC	15397	-0.47	38.25	-47.16	-465.82	-21.44	-1.24	-1114.60	-37.31	-923.11	474	-38.42	0.86
glcmClusterShadeDWI	56677	-1.10	-250.52	-73.04	-408.71	25.05	-30.27	678	353	-1693.55	1408	7499	0.41
glcmClusterShadeT2	-14.60	0.03	0.13	-0.81	9.85	1.61	0.00	0.00	-12.23	6.06	-49.48	14.92	0.06
glcmClusterTendencyADC	630	-0.02	2.18	-1.69	-17.16	-0.85	-0.10	-35.30	-2.73	-40.79	21.80	6.90	0.33
glcmClusterTendencyDWI	-3086	0.11	7.10	2.47	23.32	-0.28	1.68	-113.11	4.35	268	227	309	0.00
glcmClusterTendencyT2	9.68	0.00	0.21	-0.10	0.98	-0.39	0.00	0.00	0.29	0.95	-10.35	2.35	0.00
glcmContrastADC	129	0.00	0.42	-0.21	-2.06	-0.17	-0.06	-2.95	-0.74	-8.43	6.83	0.59	0.00
glcmContrastDWI	-288.45	0.01	0.29	1.16	4.32	0.85	0.09	-7.61	0.09	13.81	10.59	-4.95	0.00

glcmContrastT2	7.08	0.00	0.00	-0.01	0.58	-0.12	0.00	0.00	-1.00	-0.28	-1.58	-1.01	0.00
glcmCorrelationADC	0.37	0.00	0.00	0.00	-0.01	0.00	0.00	-0.01	0.00	0.03	0.02	0.10	0.00
glcmCorrelationDWI	0.40	0.00	0.00	0.00	-0.01	0.00	0.00	0.00	0.00	0.01	0.01	0.09	0.00
glcmCorrelationT2	0.43	0.00	0.00	0.00	0.00	0.00	0.00	0.00	0.03	0.02	-0.08	0.04	0.00
glcmDifferenceAverageADC	9.19	0.00	0.03	-0.01	-0.15	-0.01	0.00	-0.18	-0.05	-0.68	0.41	0.07	0.00
glcmDifferenceAverageDWI	-9.12	0.00	0.00	0.05	0.17	0.03	0.00	-0.22	0.03	0.61	0.19	0.22	0.00
glcmDifferenceAverageT2	2.05	0.00	0.01	0.00	0.04	-0.02	0.00	0.00	-0.14	-0.07	-0.29	-0.18	0.00
glcmDifferenceEntropyADC	4.86	0.00	0.02	0.00	-0.06	0.00	0.00	-0.06	-0.02	-0.37	0.15	0.15	0.00
glcmDifferenceEntropyDWI	-0.28	0.00	0.00	0.01	0.03	0.01	0.00	-0.07	0.02	0.16	0.05	0.42	0.00
glcmDifferenceEntropyT2	2.59	0.00	0.01	0.00	0.02	-0.02	0.00	0.00	-0.07	-0.06	-0.24	-0.11	0.00
glcmDifferenceVarianceADC	60.42	0.00	0.21	-0.11	-0.92	-0.09	-0.03	-1.12	-0.36	-4.06	3.37	0.90	0.00
glcmDifferenceVarianceDWI	-86.17	0.01	0.22	0.24	0.67	0.26	0.03	-3.85	0.16	3.49	7.03	8.77	0.00
glcmDifferenceVarianceT2	2.50	0.00	-0.01	0.00	0.35	-0.04	0.00	0.00	-0.35	-0.08	-0.71	-0.33	0.00
glcmIdADC	0.13	0.00	0.00	0.00	0.01	0.00	0.00	0.01	0.00	0.07	-0.03	-0.02	0.00
glcmIdDWI	0.92	0.00	0.00	0.00	-0.01	0.00	0.00	0.01	0.00	-0.02	0.01	-0.05	0.00
glcmIdmADC	0.03	0.00	0.00	0.00	0.01	0.00	0.00	0.01	0.00	0.08	-0.03	-0.02	0.00
glcmIdmDWI	0.89	0.00	0.00	0.00	-0.01	0.00	0.00	0.01	0.00	-0.02	0.02	-0.05	0.00
glcmIdmnADC	1.03	0.00	0.00	0.00	0.00	0.00	0.00	0.00	0.00	0.00	0.00	0.01	0.01
glcmIdmnDWI	1.00	0.00	0.00	0.00	0.00	0.00	0.00	0.00	0.00	0.00	0.00	0.01	0.32
glcmIdmnT2	0.99	0.00	0.00	0.00	0.00	0.00	0.00	0.00	0.00	0.00	0.00	0.00	0.23

glcmIdmT2	0.36	0.00	0.00	0.00	0.00	0.00	0.00	0.00	0.02	0.02	0.06	0.04	0.00
glcmIdnADC	1.02	0.00	0.00	0.00	0.00	0.00	0.00	0.00	0.00	0.00	0.00	0.02	0.00
glcmIdnDWI	0.97	0.00	0.00	0.00	0.00	0.00	0.00	0.00	0.00	0.00	0.01	0.02	0.32
glcmIdnT2	0.92	0.00	0.00	0.00	0.00	0.00	0.00	0.00	0.01	0.00	0.00	0.01	0.07
glcmIdT2	0.44	0.00	0.00	0.00	0.00	0.00	0.00	0.00	0.02	0.02	0.05	0.03	0.00
glcmImc1ADC	-0.03	0.00	0.00	0.00	0.00	0.00	0.00	0.00	0.00	-0.04	0.00	0.02	0.00
glcmImc1DWI	-0.21	0.00	0.00	0.00	0.00	0.00	0.00	-0.01	0.00	0.00	0.02	0.01	0.10
glcmImc1T2	-0.02	0.00	0.00	0.00	0.00	0.00	0.00	0.00	-0.01	-0.02	0.00	-0.03	0.00
glcmImc2ADC	0.45	0.00	0.00	0.00	0.00	0.00	0.00	-0.01	0.00	0.02	0.01	0.02	0.00
glcmImc2DWI	0.52	0.00	0.00	0.00	0.00	0.00	0.00	0.00	0.00	0.00	-0.02	0.04	0.00
glcmImc2T2	0.44	0.00	0.00	0.00	0.00	0.00	0.00	0.00	0.03	0.02	-0.04	0.07	0.01
glcmInverseVarianceADC	0.07	0.00	0.00	0.00	0.01	0.00	0.00	0.00	0.00	0.01	-0.02	0.02	0.00
glcmInverseVarianceDWI	0.56	0.00	0.00	0.00	0.00	0.00	0.00	0.01	0.00	-0.02	0.00	-0.01	0.00
glcmInverseVarianceT2	0.39	0.00	0.00	0.00	0.00	0.00	0.00	0.00	0.00	0.00	0.02	0.01	0.00
glcmJointAverageADC	50.19	0.00	0.26	-0.13	-1.45	-0.06	-0.01	-1.81	-0.21	-3.91	2.00	2.58	0.00
glcmJointAverageDWI	-35.08	0.00	-0.01	0.11	0.38	0.04	0.03	-1.24	0.29	5.40	1.12	4.82	0.00
glcmJointAverageT2	13.02	0.00	0.03	-0.02	0.00	-0.10	0.00	0.00	0.20	-0.07	-2.32	0.25	0.00
glcmJointEnergyADC	0.03	0.00	0.00	0.00	0.00	0.00	0.00	0.00	0.00	0.02	-0.01	-0.01	0.00
glcmJointEnergyDWI	0.06	0.00	0.00	0.00	0.00	0.00	0.00	0.00	0.00	0.00	0.01	0.00	0.00
glcmJointEnergyT2	0.00	0.00	0.00	0.00	0.00	0.00	0.00	0.00	0.00	0.00	0.02	0.01	0.00

glcmJointEntropyADC	11.70	0.00	0.05	-0.01	-0.19	0.00	0.00	-0.17	-0.05	-0.81	0.43	0.52	0.00
glcmJointEntropyDWI	1.37	0.00	0.00	0.00	0.00	0.01	0.01	-0.14	0.06	0.36	0.09	1.26	0.00
glcmJointEntropyT2	6.54	0.00	0.02	-0.01	0.02	-0.04	0.00	0.00	-0.09	-0.09	-0.79	-0.15	0.00
glcmMaximumProbabilityADC	0.02	0.00	0.00	0.00	0.01	0.00	0.00	0.00	0.00	0.04	-0.02	-0.01	0.00
glcmMaximumProbabilityDWI	0.10	0.00	0.00	0.00	0.00	0.00	0.00	0.00	0.00	0.00	0.01	-0.01	0.00
glcmMaximumProbabilityT2	0.01	0.00	0.00	0.00	0.00	0.00	0.00	0.00	0.01	0.01	0.04	0.01	0.00
glcmMCCADC	0.37	0.00	0.00	0.00	0.00	0.00	0.00	-0.01	0.00	0.03	0.01	0.04	0.00
glcmMCCDWI	0.40	0.00	0.00	0.00	0.00	0.00	0.00	0.00	0.00	0.01	-0.01	0.06	0.00
glcmMCCT2	0.37	0.00	0.00	0.00	0.00	0.00	0.00	0.00	0.03	0.02	-0.04	0.07	0.00
glcmSumAverageADC	100	0.00	0.52	-0.27	-2.90	-0.12	-0.02	-3.61	-0.42	-7.81	4.00	5.16	0.00
glcmSumAverageDWI	-70.15	0.00	-0.03	0.21	0.75	0.09	0.06	-2.48	0.58	10.80	2.25	9.65	0.00
glcmSumAverageT2	26.03	0.00	0.06	-0.04	0.00	-0.21	0.00	0.00	0.40	-0.13	-4.63	0.49	0.00
glcmSumEntropyADC	6.87	0.00	0.03	-0.01	-0.11	0.00	0.00	-0.11	-0.03	-0.40	0.26	0.31	0.00
glcmSumEntropyDWI	1.49	0.00	0.00	0.00	-0.01	0.00	0.00	-0.06	0.03	0.23	0.02	0.76	0.00
glcmSumEntropyT2	4.01	0.00	0.01	0.00	0.01	-0.02	0.00	0.00	-0.02	-0.01	-0.45	-0.01	0.00
glcmSumSquaresADC	190	-0.01	0.65	-0.48	-4.80	-0.26	-0.04	-9.56	-0.87	-12.30	7.16	1.87	0.17
glcmSumSquaresDWI	-843.64	0.03	1.85	0.91	6.91	0.14	0.44	-30.18	1.11	70.39	59.30	76.05	0.00
glcmSumSquaresT2	4.19	0.00	0.05	-0.03	0.39	-0.13	0.00	0.00	-0.18	0.17	-2.98	0.33	0.00
gldmDependenceEntropyADC	8.80	0.00	0.02	-0.01	-0.08	0.00	0.00	-0.07	-0.02	-0.28	0.17	0.40	0.00
gldmDependenceEntropyDWI	6.76	0.00	0.00	-0.01	-0.04	0.00	0.00	-0.03	0.02	0.14	0.06	0.64	0.00

gldmDependenceEntropyT2	6.43	0.00	0.00	0.00	0.00	-0.01	0.00	0.00	0.07	0.08	-0.28	0.14	0.00
gldmDependenceNonUniformityADC	5910	-0.33	115.83	-9.48	-1081.1	-37.26	-1.85	305	56.94	738	30.06	-3622	0.58
gldmDependenceNonUniformityDWI	-8066.72	-0.45	182.97	-5.59	-1163.1	-35.11	8.29	317	67.95	1208	-166	-2433	0.46
gldmDependenceNonUniformityNormalizedADC	0.21	0.00	0.00	0.00	0.00	0.00	0.00	0.00	0.00	-0.01	0.01	-0.01	0.00
gldmDependenceNonUniformityNormalizedDWI	-0.14	0.00	0.00	0.00	0.00	0.00	0.00	0.00	0.00	0.01	0.00	0.00	0.00
gldmDependenceNonUniformityNormalizedT2	0.12	0.00	0.00	0.00	0.00	0.00	0.00	0.00	-0.01	-0.01	-0.01	-0.01	0.00
gldmDependenceNonUniformityT2	-27049	0.19	136	-62.66	-208.7	-68.15	0.00	0.00	6549	3761	-6098	5720	0.00
gldmDependenceVarianceADC	-15.38	0.00	-0.28	-0.15	0.95	-0.08	0.04	-0.15	0.20	5.78	-1.83	0.97	0.00
gldmDependenceVarianceDWI	28.44	0.00	-0.02	-0.07	-0.16	-0.06	-0.01	0.12	-0.08	1.07	1.49	-2.55	0.00
gldmDependenceVarianceT2	5.87	0.00	-0.17	0.03	0.35	0.27	0.00	0.00	2.90	1.65	2.81	4.05	0.00
gldmGrayLevelNonUniformityADC	-6046.92	-0.49	77.71	15.00	-1289.5	-53.89	9.32	1470	89.58	2164	-1329	-7910	0.74
gldmGrayLevelNonUniformityDWI	7009	-0.11	26.33	-16.12	-457.7	-14.85	1.77	397	-102	-534	407	-3094	0.85
gldmGrayLevelNonUniformityT2	-25270	0.16	-93.05	3.62	414.9	449	0.00	0.00	5955	4494	-3877	5322	0.01
gldmGrayLevelVarianceADC	220	-0.01	0.71	-0.52	-5.14	-0.30	-0.06	-9.82	-1.01	-14.10	8.58	3.05	0.06
gldmGrayLevelVarianceDWI	-859.76	0.03	1.84	0.90	7.42	0.17	0.46	-32.45	1.12	72.59	60.13	83.60	0.00
gldmGrayLevelVarianceT2	4.24	0.00	0.04	-0.02	0.62	-0.13	0.00	0.00	-0.31	0.16	-3.31	0.27	0.00
gldmHighGrayLevelEmphasisADC	1804	-0.05	7.66	-4.99	-50.05	-2.52	-0.44	-82.95	-7.67	-128.38	65.02	50.37	0.00
gldmHighGrayLevelEmphasisDWI	-3208.75	0.06	6.27	5.01	18.72	0.15	1.83	-127.27	9.13	347	202	287	0.00

gldmHighGrayLevelEmphasisT2	153	0.00	0.52	-0.37	1.52	-2.00	0.00	0.00	6.38	0.27	-43.10	5.67	0.00
gldmLargeDependenceEmphasisADC	-17.30	0.02	-1.79	-0.65	2.64	-0.64	0.14	0.31	1.50	37.95	-3.40	-13.70	0.00
gldmLargeDependenceEmphasisDWI	162	0.00	0.06	-0.34	-1.23	-0.32	-0.07	2.15	-1.23	-2.28	7.41	-19.07	0.00
gldmLargeDependenceEmphasisT2	1.16	0.00	-0.75	0.20	1.02	1.59	0.00	0.00	11.81	9.57	15.29	14.14	0.00
gldmLargeDependenceHighGrayLevelEmphasisADC	33169	-1.46	329.36	-218.32	-1891	-105	14	-2664	-180	-3074	1805	2193	0.00
gldmLargeDependenceHighGrayLevelEmphasisDWI	-13095.81	-0.20	-73.12	27.86	-206	-107	-3	-291	436	13993	621	414	0.00
gldmLargeDependenceHighGrayLevelEmphasisT2	5799	-0.49	7.30	-18.16	-133	-45	0	0	1046	455	-1414	1373	0.01
gldmLargeDependenceLowGrayLevelEmphasisADC	2.40	0.00	-0.37	0.03	0.87	-0.04	0.00	0.98	0.17	5.20	-0.31	-3.04	0.00
gldmLargeDependenceLowGrayLevelEmphasisDWI	4.13	0.00	0.05	-0.03	-0.17	-0.03	0.00	-0.08	-0.04	-0.60	0.56	-0.14	0.02
gldmLargeDependenceLowGrayLevelEmphasisT2	-2.69	0.00	-0.04	0.02	0.11	0.09	0.00	0.00	0.73	0.45	1.02	0.31	0.00
gldmLowGrayLevelEmphasisADC	-0.06	0.00	0.00	0.00	0.01	0.00	0.00	0.01	0.00	0.02	-0.01	-0.02	0.00
gldmLowGrayLevelEmphasisDWI	0.06	0.00	0.00	0.00	0.00	0.00	0.00	0.00	0.00	0.00	0.01	0.00	0.00
gldmLowGrayLevelEmphasisT2	-0.01	0.00	0.00	0.00	0.00	0.00	0.00	0.00	0.00	0.00	0.01	0.00	0.02
gldmSmallDependenceEmphasisADC	0.38	0.00	0.00	0.00	-0.01	0.00	0.00	-0.01	0.00	-0.03	0.02	0.03	0.00
gldmSmallDependenceEmphasisDWI	-0.51	0.00	0.00	0.00	0.01	0.00	0.00	-0.01	0.00	0.03	0.01	0.04	0.00
gldmSmallDependenceEmphasisT2	0.16	0.00	0.00	0.00	0.01	0.00	0.00	0.00	0.00	-0.01	-0.03	0.00	0.00
gldmSmallDependenceHighGrayLevelEmphasisADC	412	-0.01	1.07	-0.96	-9.23	-0.53	-0.13	-16.89	-1.44	-26.43	15.70	7.42	0.02
gldmSmallDependenceHighGrayLevelEmphasisDWI	-1541.32	0.05	3.70	3.18	14.03	0.61	0.70	-66.06	0.16	97.34	111	50.66	0.00

gldmSmallDependenceHighGrayLevelEmphasisT2	20.92	0.00	-0.03	-0.01	1.93	-0.23	0.00	0.00	0.04	-1.04	-8.09	-0.37	0.00
gldmSmallDependenceLowGrayLevelEmphasisADC	-0.01	0.00	0.00	0.00	0.00	0.00	0.00	0.00	0.00	0.00	0.00	0.00	0.02
gldmSmallDependenceLowGrayLevelEmphasisDWI	0.00	0.00	0.00	0.00	0.00	0.00	0.00	0.00	0.00	0.00	0.00	0.00	0.02
gldmSmallDependenceLowGrayLevelEmphasisT2	0.00	0.00	0.00	0.00	0.00	0.00	0.00	0.00	0.00	0.00	0.00	0.00	0.01
glrlmGrayLevelNonUniformityADC	-165	-0.28	70.03	1.97	-705	-9.93	2.97	611	47.57	108	-612.07	-2740.94	0.85
glrlmGrayLevelNonUniformityDWI	4509	-0.12	25.82	-10.05	-329	-9.76	1.10	252	-61.10	-363	201	-1930.50	0.87
glrlmGrayLevelNonUniformityNormalizedADC	0.04	0.00	0.00	0.00	0.00	0.00	0.00	0.01	0.00	0.02	-0.01	-0.02	0.00
glrlmGrayLevelNonUniformityNormalizedDWI	0.17	0.00	0.00	0.00	0.00	0.00	0.00	0.00	0.00	-0.01	0.01	-0.02	0.00
glrlmGrayLevelNonUniformityNormalizedT2	0.11	0.00	0.00	0.00	0.00	0.00	0.00	0.00	0.00	0.00	0.04	0.00	0.00
glrlmGrayLevelNonUniformityT2	-17542	0.15	-14.16	-12.83	119.22	233	0.00	0.00	4102	2994	-3508	3814	0.01
glrlmGrayLevelVarianceADC	235	-0.01	0.76	-0.56	-5.47	-0.33	-0.06	-10.39	-1.03	-14.88	9.00	3.98	0.04
glrlmGrayLevelVarianceDWI	-818.35	0.03	1.80	0.87	6.91	0.21	0.43	-32.25	1.14	68.50	59.06	85.03	0.00
glrlmGrayLevelVarianceT2	4.25	0.00	0.04	-0.02	0.71	-0.13	0.00	0.00	-0.30	0.20	-3.47	0.42	0.00
glrlmHighGrayLevelRunEmphasisADC	1841	-0.05	7.91	-5.18	-50.97	-2.64	-0.45	-84.45	-7.88	-130.41	65.23	55.86	0.00
glrlmHighGrayLevelRunEmphasisDWI	-3252.98	0.06	6.53	4.94	18.70	0.20	1.86	-129.21	9.27	343	210	296	0.00
glrlmHighGrayLevelRunEmphasisT2	153	0.00	0.51	-0.37	1.84	-2.02	0.00	0.00	6.30	0.43	-43.44	6.13	0.00
glrlmLongRunEmphasisADC	12.22	0.00	-0.25	-0.07	-0.32	-0.14	0.00	0.12	0.15	5.79	0.59	-5.35	0.00
glrlmLongRunEmphasisDWI	10.75	0.00	0.02	-0.02	-0.10	-0.02	0.00	0.23	-0.13	-0.32	0.50	-2.00	0.00
glrlmLongRunEmphasisT2	-0.94	0.00	-0.03	0.01	0.04	0.05	0.00	0.00	0.62	0.51	0.44	0.44	0.00

glrlmLongRunHighGrayLevelEmphasisADC	3369	-0.08	16.22	-17.00	-152.34	-11.10	0.58	-193.63	-17.02	-125.99	183	-85.49	0.00
glrlmLongRunHighGrayLevelEmphasisDWI	-3589.68	0.07	2.23	6.60	8.81	-3.95	1.89	-108.36	10.87	820	268	-59.80	0.00
glrlmLongRunHighGrayLevelEmphasisT2	227	-0.02	0.61	-0.75	-1.98	-3.08	0.00	0.00	33.51	19.84	-69.41	37.01	0.00
glrlmLongRunLowGrayLevelEmphasisADC	0.94	0.00	-0.04	0.00	0.03	-0.01	0.00	0.14	0.03	0.78	0.09	-0.62	0.00
glrlmLongRunLowGrayLevelEmphasisDWI	0.36	0.00	0.00	0.00	-0.01	0.00	0.00	0.01	-0.01	-0.05	0.04	-0.06	0.00
glrlmLongRunLowGrayLevelEmphasisT2	-0.16	0.00	0.00	0.00	0.00	0.00	0.00	0.00	0.04	0.02	0.04	0.02	0.00
glrlmLowGrayLevelRunEmphasisADC	-0.05	0.00	0.00	0.00	0.01	0.00	0.00	0.01	0.00	0.02	-0.01	-0.02	0.00
glrlmLowGrayLevelRunEmphasisDWI	0.07	0.00	0.00	0.00	0.00	0.00	0.00	0.00	0.00	0.00	0.01	0.00	0.00
glrlmLowGrayLevelRunEmphasisT2	0.00	0.00	0.00	0.00	0.00	0.00	0.00	0.00	0.00	0.00	0.01	0.00	0.03
glrlmRunEntropyADC	6.89	0.00	0.01	-0.01	-0.09	-0.01	0.00	-0.09	-0.02	-0.12	0.18	0.19	0.00
glrlmRunEntropyDWI	3.20	0.00	0.00	0.00	-0.01	0.00	0.00	-0.04	0.02	0.19	0.07	0.51	0.00
glrlmRunEntropyT2	3.81	0.00	0.00	0.00	0.02	-0.01	0.00	0.00	0.05	0.06	-0.27	0.09	0.00
glrlmRunLengthNonUniformityADC	31661	-2.64	934	-69.76	-5456	-24.88	-14.45	547	219	-2947	748	-5741	0.69
glrlmRunLengthNonUniformityDWI	-32961	-2.76	1137	-34.28	-7199	-161	26.84	2037	711	6445	-2297	-13042	0.50
glrlmRunLengthNonUniformityNormalizedADC	0.98	0.00	0.00	0.00	-0.01	0.00	0.00	-0.01	0.00	-0.07	0.02	0.03	0.00
glrlmRunLengthNonUniformityNormalizedDWI	0.30	0.00	0.00	0.00	0.01	0.00	0.00	-0.01	0.00	0.02	-0.02	0.05	0.00
glrlmRunLengthNonUniformityNormalizedT2	0.83	0.00	0.00	0.00	0.00	0.00	0.00	0.00	-0.02	-0.02	-0.04	-0.03	0.00
glrlmRunLengthNonUniformityT2	-169902	1.44	835	-407	-1253	-472	0.00	0.00	42427	23836	-37582	37544	0.00
glrlmRunPercentageADC	1.01	0.00	0.00	0.00	-0.01	0.00	0.00	-0.01	0.00	-0.06	0.01	0.03	0.00

glrlmRunPercentageDWI	0.47	0.00	0.00	0.00	0.00	0.00	0.00	-0.01	0.00	0.01	-0.01	0.05	0.00
glrlmRunPercentageT2	0.91	0.00	0.00	0.00	0.00	0.00	0.00	0.00	-0.02	-0.02	-0.03	-0.03	0.00
glrlmRunVarianceADC	4.00	0.00	-0.12	-0.04	-0.20	-0.08	0.00	-0.02	0.09	3.43	0.44	-3.12	0.00
glrlmRunVarianceDWI	4.12	0.00	0.01	-0.01	-0.06	-0.01	0.00	0.10	-0.06	-0.10	0.26	-1.09	0.00
glrlmRunVarianceT2	-1.01	0.00	-0.01	0.00	0.03	0.02	0.00	0.00	0.33	0.25	0.19	0.24	0.00
glrlmShortRunEmphasisADC	0.97	0.00	0.00	0.00	0.00	0.00	0.00	-0.01	0.00	-0.05	0.01	0.04	0.00
glrlmShortRunEmphasisDWI	0.54	0.00	0.00	0.00	0.00	0.00	0.00	-0.01	0.00	0.01	-0.01	0.04	0.00
glrlmShortRunEmphasisT2	0.95	0.00	0.00	0.00	0.00	0.00	0.00	0.00	-0.01	-0.02	-0.03	-0.02	0.00
glrlmShortRunHighGrayLevelEmphasisADC	1718	-0.05	6.99	-4.62	-45.71	-2.37	-0.46	-77.18	-7.18	-120.73	59.90	50.64	0.00
glrlmShortRunHighGrayLevelEmphasisDWI	-3177.20	0.06	6.53	4.86	18.97	0.34	1.80	-126.90	8.43	314	206	282	0.00
glrlmShortRunHighGrayLevelEmphasisT2	135	0.00	0.44	-0.31	2.23	-1.82	0.00	0.00	4.22	-0.47	-38.71	3.71	0.00
glrlmShortRunLowGrayLevelEmphasisADC	-0.06	0.00	0.00	0.00	0.00	0.00	0.00	0.00	0.00	0.01	-0.01	-0.01	0.00
glrlmShortRunLowGrayLevelEmphasisDWI	0.05	0.00	0.00	0.00	0.00	0.00	0.00	0.00	0.00	0.00	0.01	0.00	0.00
glrlmShortRunLowGrayLevelEmphasisT2	0.00	0.00	0.00	0.00	0.00	0.00	0.00	0.00	0.00	0.00	0.01	0.00	0.17
glszmGrayLevelNonUniformityADC	424	-0.01	1.27	-0.28	-11.79	0.42	-0.25	6.91	0.15	-42.72	14.31	63.34	0.04
glszmGrayLevelNonUniformityDWI	-149.43	-0.01	2.94	-0.80	-16.62	-0.43	0.20	-7.87	4.08	17.24	0.60	118	0.00
glszmGrayLevelNonUniformityNormalizedADC	-0.03	0.00	0.00	0.00	0.01	0.00	0.00	0.01	0.00	0.02	-0.01	-0.02	0.00
glszmGrayLevelNonUniformityNormalizedDWI	0.14	0.00	0.00	0.00	0.00	0.00	0.00	0.00	0.00	0.00	0.00	-0.02	0.00
glszmGrayLevelNonUniformityNormalizedT2	0.08	0.00	0.00	0.00	0.00	0.00	0.00	0.00	0.00	0.00	0.02	0.00	0.00

glszmGrayLevelNonUniformityT2	-220	-0.01	0.78	-0.85	8.96	4.82	0.00	0.00	128	75.54	-251	137	0.11
glszmGrayLevelVarianceADC	374	-0.01	1.21	-0.97	-9.16	-0.54	-0.10	-15.92	-1.27	-22.79	12.74	12.98	0.00
glszmGrayLevelVarianceDWI	-498	0.02	1.40	0.54	3.08	0.43	0.27	-29.45	1.14	41.02	53.54	93.55	0.00
glszmGrayLevelVarianceT2	3.01	0.00	0.00	-0.03	1.63	-0.06	0.00	0.00	0.10	0.59	-6.42	1.45	0.00
glszmHighGrayLevelZoneEmphasisADC	2656	-0.07	10.00	-7.07	-66.30	-3.97	-0.75	-110.20	-10.04	-175.76	83.53	92.73	0.00
glszmHighGrayLevelZoneEmphasisDWI	-3364.47	0.08	8.87	4.71	13.45	0.86	1.85	-129.84	10.56	309	273	373	0.00
glszmHighGrayLevelZoneEmphasisT2	158	0.00	0.33	-0.43	6.19	-2.18	0.00	0.00	3.09	2.44	-51.10	10.84	0.00
glszmLargeAreaEmphasisADC	-3781912	-48.32	-27324	38511	-231395	-38965	-3078	499651	89103	2720331	-232836	-4545718	0.12
glszmLargeAreaEmphasisDWI	1523949	62.17	-4547	-7633	-39174	-5183	436	3003	-19153.79	-95039.96	97937	-221506	0.13
glszmLargeAreaEmphasisT2	-1080907	6	-5941	2529	27812	17786	0.00	0.00	171961	134137	18748	88720	0.00
glszmLargeAreaHighGrayLevelEmphasisADC	-2.97E+08	-3.82E+04	4.98E+05	2.87E+06	-1.81E+07	-1.19E+06	-2.19E+04	4.16E+07	3.36E+06	1.03E+08	-3.59E+07	-2.68E+08	0.61
glszmLargeAreaHighGrayLevelEmphasisDWI	1.48E+07	-1.42E+04	-4.98E+05	4.71E+05	-4.24E+06	-7.78E+04	1.08E+05	1.87E+07	-2.99E+06	-8.23E+05	-2.71E+06	-9.37E+07	0.83
glszmLargeAreaHighGrayLevelEmphasisT2	-1.E+08	910	3.E+05	-1.E+05	-7.E+05	2.E+04	0.00	0.00	2.E+07	1.E+07	-1.E+07	2.E+07	0.00
glszmLargeAreaLowGrayLevelEmphasisADC	-50227	33.99	-3308	1263	-6492	-3144	-447	3782	8457	203110	30629	-187099	0.00
glszmLargeAreaLowGrayLevelEmphasisDWI	22783	1.03	18.26	-99.66	-528	-82.04	-4.33	-329	-180	-1097	1299	-1754	0.01
glszmLargeAreaLowGrayLevelEmphasisT2	-29223	0.26	-277.37	119.06	1303	630	0.00	0.00	4602	3366	2340	1019	0.00

glszmLowGrayLevelZoneEmphasisADC	-0.02	0.00	0.00	0.00	0.01	0.00	0.00	0.01	0.00	0.01	-0.01	-0.01	0.00
glszmLowGrayLevelZoneEmphasisDWI	0.13	0.00	0.00	0.00	0.00	0.00	0.00	0.00	0.00	-0.01	0.01	-0.01	0.00
glszmLowGrayLevelZoneEmphasisT2	0.00	0.00	0.00	0.00	0.00	0.00	0.00	0.00	0.00	0.00	0.01	0.00	0.15
glszmSizeZoneNonUniformityADC	5580	-0.10	9.63	-10.79	-128.16	-2.00	-2.34	-91.47	-4.56	-418.88	170	505	0.02
glszmSizeZoneNonUniformityDWI	-8973.64	0.11	59.89	-0.58	-103.19	-8.95	4.23	-281.03	47.34	749	504	1940	0.00
glszmSizeZoneNonUniformityNormalizedADC	0.54	0.00	0.00	0.00	-0.01	0.00	0.00	0.00	0.00	-0.03	0.03	0.06	0.00
glszmSizeZoneNonUniformityNormalizedDWI	-0.41	0.00	0.00	0.00	0.01	0.00	0.00	-0.02	0.00	0.04	0.02	0.08	0.00
glszmSizeZoneNonUniformityNormalizedT2	0.30	0.00	0.00	0.00	0.02	0.00	0.00	0.00	0.04	-0.02	-0.05	0.04	0.00
glszmSizeZoneNonUniformityT2	-1587.31	0.06	6.37	-2.40	104.43	3.58	0.00	0.00	701	268	-1217.33	775	0.11
glszmSmallAreaEmphasisADC	0.89	0.00	0.00	0.00	-0.01	0.00	0.00	-0.01	0.00	-0.04	0.04	0.10	0.00
glszmSmallAreaEmphasisDWI	-0.33	0.00	0.00	0.00	0.01	0.00	0.00	-0.02	0.01	0.04	0.02	0.13	0.00
glszmSmallAreaEmphasisT2	0.59	0.00	0.00	0.00	0.02	0.00	0.00	0.00	0.05	-0.02	-0.05	0.05	0.00
glszmSmallAreaHighGrayLevelEmphasisADC	1587	-0.04	5.01	-3.80	-36.06	-2.20	-0.53	-62.74	-5.44	-102.01	55.25	55.42	0.00
glszmSmallAreaHighGrayLevelEmphasisDWI	-2354.73	0.07	6.05	3.65	14.32	0.90	1.18	-96.13	5.84	190	202	275	0.00
glszmSmallAreaHighGrayLevelEmphasisT2	92.25	0.01	-0.13	-0.17	6.74	-0.97	0.00	0.00	6.06	-0.35	-35.06	10.19	0.00
glszmSmallAreaLowGrayLevelEmphasisADC	-0.02	0.00	0.00	0.00	0.00	0.00	0.00	0.00	0.00	0.00	0.00	0.00	0.01
glszmSmallAreaLowGrayLevelEmphasisDWI	0.02	0.00	0.00	0.00	0.00	0.00	0.00	0.00	0.00	0.00	0.00	0.00	0.00
glszmSmallAreaLowGrayLevelEmphasisT2	0.00	0.00	0.00	0.00	0.00	0.00	0.00	0.00	0.00	0.00	0.01	0.00	0.02

glszmZoneEntropyADC	10.21	0.00	0.03	-0.02	-0.17	-0.01	0.00	-0.19	-0.03	-0.28	0.26	0.49	0.00
glszmZoneEntropyDWI	7.11	0.00	0.01	-0.01	-0.07	0.00	0.00	-0.07	0.00	0.05	0.07	0.27	0.01
glszmZoneEntropyT2	6.06	0.00	0.01	-0.01	-0.06	-0.01	0.00	0.00	-0.29	0.11	-0.15	-0.25	0.00
glszmZonePercentageADC	0.42	0.00	0.00	0.00	-0.01	0.00	0.00	-0.01	0.00	-0.03	0.02	0.03	0.00
glszmZonePercentageDWI	-0.66	0.00	0.00	0.00	0.01	0.00	0.00	-0.02	0.00	0.04	0.01	0.05	0.00
glszmZonePercentageT2	0.16	0.00	0.00	0.00	0.01	0.00	0.00	0.00	0.00	-0.01	-0.03	0.00	0.00
glszmZoneVarianceADC	-3.83E+06	-6.22E+01	-2.59E+04	3.85E+04	-2.28E+05	-3.78E+04	-2.96E+03	5.02E+05	8.66E+04	2.66E+06	-2.45E+05	-4.49E+06	0.13
glszmZoneVarianceDWI	1.49E+06	60.90	-4.56E+03	-7.51E+03	-3.87E+04	-5.08E+03	446	2.88E+03	-1.87E+04	-9.28E+04	9.81E+04	-2.17E+05	0.15
glszmZoneVarianceT2	-1077603	6.06	-5930	2521	27773	17753	0	0	171507	133762	18606	88521	0.00
ngtdmBusynessADC	2.66	0.00	-0.57	0.06	-0.40	-0.09	0.02	4.64	-0.04	7.38	-1.90	-16.71	0.12
ngtdmBusynessDWI	48.93	0.00	0.10	-0.13	-0.92	-0.11	-0.02	0.16	-0.42	-3.01	2.23	-4.11	0.00
ngtdmBusynessT2	-40.64	0.00	-0.27	0.11	0.71	1.37	0.00	0.00	5.66	7.60	-2.47	3.34	0.01
ngtdmCoarsenessADC	-0.01	0.00	0.00	0.00	0.00	0.00	0.00	0.00	0.00	0.00	0.00	0.00	0.42
ngtdmCoarsenessDWI	-0.02	0.00	0.00	0.00	0.00	0.00	0.00	0.00	0.00	0.00	0.00	0.00	0.52
ngtdmCoarsenessT2	0.00	0.00	0.00	0.00	0.00	0.00	0.00	0.00	0.00	0.00	0.00	0.00	0.35
ngtdmComplexityADC	15549	-0.24	46.75	-39.21	-340	-23.25	-5.56	-640	-66.41	-942	813	214	0.00
ngtdmComplexityDWI	-34779	1.62	160	47.64	-65.84	29.66	15.04	-1939	34.17	1943	4100	3979	0.00
ngtdmComplexityT2	177	0.08	-1.26	-1.79	47.46	-2.28	0.00	0.00	60.84	27.74	-239	63.88	0.00

ngtdmContrastADC	0.15	0.00	0.00	0.00	0.00	0.00	0.00	0.00	0.00	-0.02	0.02	-0.02	0.00
ngtdmContrastDWI	-1.00	0.00	0.00	0.00	0.02	0.00	0.00	-0.01	0.00	0.05	-0.01	-0.11	0.00
ngtdmContrastT2	0.03	0.00	0.00	0.00	0.00	0.00	0.00	0.00	-0.01	0.00	0.00	0.00	0.03
ngtdmStrengthADC	3.54	0.00	0.00	0.00	-0.05	0.00	0.00	-0.15	-0.01	-0.20	0.16	0.11	0.38
ngtdmStrengthDWI	1.48	0.00	-0.03	0.02	0.13	0.02	0.00	-0.24	0.06	-0.01	0.08	2.30	0.00
ngtdmStrengthT2	-0.04	0.00	0.00	0.00	0.03	0.00	0.00	0.00	0.02	-0.01	-0.01	0.04	0.04

Supplementary Table 3. Total number of sequenced reads, unique reads, covered bases and coverage per base

Sample ID	Total number of sequenced reads	Total number of uniquely mapped non-duplicate reads[a]	Total number of covered bases (Mb)[b]	Median coverage per base[b]	Minimum coverage per base[b]	Maximum coverage per base[b]	Percentage of targeted bases with coverage ≥30[b,c,d]
T_001	169600000	110017870	11040.4	117.0	0	4773	99.0%
T_002	168000000	110679618	11103.0	115.0	0	2632	99.0%
T_003	183000000	118643264	11890.7	125.0	0	2694	99.0%
T_004	200000000	127458930	12797.0	134.0	0	3175	99.0%
T_005	165000000	108306450	10873.3	116.0	0	2615	99.0%
T_006	191600000	123306116	12359.3	129.0	0	2383	99.0%
T_007	199200000	126689030	12702.1	134.0	0	2955	99.0%
T_008	196400000	123106042	12324.4	129.0	0	3112	99.0%
T_009	203800000	130482752	13081.0	138.0	0	2446	99.0%
T_010	174400000	113401606	11380.7	121.0	0	2683	99.0%
T_011	198800000	127301438	12751.2	137.0	0	2866	99.0%
T_012	203400000	129176532	12962.0	140.0	0	2988	99.0%
T_013	216600000	136701680	13711.4	147.0	0	3085	99.0%
T_014	181800000	120305458	12073.3	129.0	0	2836	99.0%
T_015	163200000	107128796	10745.5	115.0	0	2800	99.0%
T_016	178200000	113999368	11438.6	122.0	0	2738	99.0%
T_017	183600000	124211754	12436.7	127.0	0	3595	99.0%
T_018	203400000	134600872	13456.6	142.0	0	2822	99.0%
T_019	172600000	116187898	11644.5	122.0	0	2664	99.0%
T_020	182200000	120234622	12049.2	127.0	0	2352	99.0%
T_021	210200000	136447406	13684.9	139.0	0	3025	99.0%
T_022	164600000	111760200	11198.7	117.0	0	2985	99.0%
T_023	188600000	126147470	12655.4	133.0	0	3324	99.0%
T_024	171800000	117133020	11746.8	123.0	0	2403	99.0%
T_025	196600000	134752866	13495.8	143.0	0	2965	99.0%
T_026	241200000	154958410	15526.1	161.0	0	3509	99.0%
T_027	179600000	124554334	12481.0	128.0	0	2505	99.0%
T_028	187400000	127697606	12810.2	133.0	0	2879	99.0%
T_029	200400000	138662390	13922.2	147.0	0	3358	99.0%
T_030	196000000	134378194	13474.7	132.0	0	3769	99.0%
T_031	198200000	135821092	13608.9	138.0	0	3225	99.0%
T_032	194200000	134602558	13485.1	140.0	0	3165	99.0%
T_033	228600000	144664250	14520.3	152.0	0	3923	99.0%
T_034	233200000	148220172	14874.9	158.0	0	3388	99.0%

T_035	212000000	135444592	13603.7	143.0	0	3198	99.0%
T_036	216600000	139919874	14032.1	148.0	0	3288	99.0%
T_037	209600000	134680836	13525.4	143.0	0	3408	99.0%
T_038	195400000	127902098	12840.5	131.0	0	2555	99.0%
T_039	236200000	144312316	14488.3	152.0	0	3519	99.0%
T_040	197000000	127271692	12776.4	136.0	0	2899	99.0%
T_041	214200000	138653710	13936.1	142.0	0	3444	99.0%
T_042	229800000	149497846	15024.0	152.0	0	4242	99.0%
T_043	206800000	135137030	13574.8	136.0	0	3815	99.0%
T_044	188600000	125706200	12638.4	124.0	0	2863	99.0%
T_045	198600000	133914954	13457.3	134.0	0	3240	99.0%
T_046	198200000	131075234	13173.2	136.0	0	3366	99.0%
T_047	200000000	137953264	13864.3	139.0	0	3841	99.0%
T_048	220200000	140855838	14144.6	143.0	0	3548	99.0%
T_049	215400000	143900282	14450.3	137.0	0	3741	99.0%
T_050	232400000	151221634	15191.4	144.0	0	3755	99.0%
T_051	194200000	132790508	13338.7	125.0	0	3259	99.0%
T_052	229600000	151089920	15167.8	141.0	0	3990	99.0%
T_053	230200000	154875352	15564.5	149.0	0	4106	99.0%
T_054	230200000	147943904	14858.0	132.0	0	5514	99.0%
T_055	186400000	128148624	12861.6	121.0	0	3646	99.0%
T_056	228000000	148965838	14951.6	141.0	0	3479	99.0%
T_057	222400000	150455170	15115.3	145.0	0	3261	99.0%
T_058	192800000	134842330	13560.2	130.0	0	3559	99.0%
T_059	224000000	151819424	15260.4	143.0	0	3976	99.0%
T_060	220400000	145850496	14657.0	138.0	0	4061	99.0%
T_061	203800000	138659540	13941.9	131.0	0	3478	99.0%
T_062	198600000	138587462	13906.4	132.0	0	3331	99.0%
T_063	201600000	137285790	13797.0	133.0	0	3833	99.0%
T_064	208200000	143024052	14370.2	137.0	0	3370	99.0%
T_065	210200000	133203104	13385.8	130.0	0	3203	99.0%
T_066	222400000	141011524	14168.1	135.0	0	3911	99.0%
T_067	202600000	129025618	12969.2	128.0	0	3157	99.0%
T_068	229800000	146906172	14766.2	147.0	0	3427	99.0%
T_069	210000000	136790902	13747.1	134.0	0	3452	99.0%
T_070	212600000	137222510	13775.4	136.0	0	2822	99.0%
T_071	217200000	138697614	13945.6	134.0	0	2964	99.0%
T_072	204400000	131754672	13224.7	128.0	0	3495	99.0%
T_073	205000000	135307520	13588.9	134.0	0	3681	99.0%
T_074	189400000	123665818	12422.8	122.0	0	3169	99.0%
T_075	201000000	134447212	13494.1	133.0	0	3680	99.0%

T_076	172400000	116111036	11659.5	114.0	0	2769	99.0%
T_077	182800000	122638820	12315.4	121.0	0	2852	99.0%
T_078	179200000	119182970	11960.0	117.0	0	2719	99.0%
T_079	169400000	113793812	11432.6	110.0	0	2820	99.0%
T_080	192600000	126132452	12660.6	123.0	0	2782	99.0%
T_081	170800000	120428180	12066.7	121.0	0	3536	99.0%
T_082	180400000	126019104	12590.9	129.0	0	3114	99.0%
T_083	162200000	114276034	11449.0	114.0	0	3801	99.0%
T_084	176400000	121996642	12221.3	123.0	0	2470	99.0%
T_085	168800000	118489876	11862.2	117.0	0	3218	99.0%
T_086	199800000	135633000	13579.2	136.0	0	3576	99.0%
T_087	253600000	158775516	15905.1	156.0	0	6627	99.0%
T_088	159800000	112448938	11268.7	112.0	0	2989	99.0%
T_089	218800000	143000914	14324.1	143.0	0	3533	99.0%
T_090	205000000	134447504	13474.2	133.0	0	3328	99.0%
T_091	196400000	133809434	13402.5	130.0	0	3612	99.0%
T_092	188200000	128890670	12887.7	125.0	0	3136	99.0%
T_093	164000000	115805900	11591.9	115.0	0	2807	99.0%
T_094	190200000	129754692	12978.2	128.0	0	4321	99.0%
T_095	176400000	121905918	12204.1	119.0	0	3607	99.0%
T_096	196800000	132049776	13215.2	131.0	0	3259	99.0%
N_001	110200000	76690606	7688.0	82.0	0	2077	99.0%
N_002	98400000	70530462	7067.0	76.0	0	1860	98.0%
N_003	96600000	68545302	6868.4	72.0	0	1384	99.0%
N_004	93800000	67799408	6786.6	73.0	0	1698	99.0%
N_005	127200000	85026502	8525.8	90.0	0	2325	99.0%
N_006	92200000	66514522	6663.8	71.0	0	1323	99.0%
N_007	118400000	82530358	8266.4	89.0	0	2273	98.0%
N_008	103400000	73679224	7374.9	79.0	0	1971	97.0%
N_009	91600000	65722498	6583.9	69.0	0	1350	99.0%
N_010	108600000	75574066	7569.6	79.0	0	1759	99.0%
N_011	113400000	81175626	8133.7	85.0	0	2211	98.0%
N_012	100000000	70092172	7017.5	75.0	0	1770	99.0%
N_013	110400000	75176846	7534.2	80.0	0	1839	99.0%
N_014	112200000	78358826	7854.8	83.0	0	1906	99.0%
N_015	91000000	63545944	6368.9	67.0	0	1685	99.0%
N_016	91800000	65857092	6596.5	70.0	0	1663	99.0%
N_017	87000000	62389988	6250.1	66.0	0	1547	99.0%
N_018	100600000	69545786	6973.1	74.0	0	1634	99.0%
N_019	99600000	70226170	7044.3	75.0	0	1694	99.0%
N_020	97000000	69157374	6938.6	74.0	0	1460	99.0%

N_021	110800000	76755234	7695.4	81.0	0	1818	99.0%
N_022	99600000	69107514	6921.2	73.0	0	1654	99.0%
N_023	96000000	67600640	6779.7	71.0	0	1674	99.0%
N_024	103800000	72976592	7318.2	76.0	0	1618	99.0%
N_025	111800000	94807756	7367.5	77.0	0	2181	99.0%
N_026	94000000	79919110	8025.0	86.0	0	2093	99.0%
N_027	108400000	90379644	8233.4	88.0	0	1975	99.0%
N_028	85200000	72333346	7067.5	75.0	0	1501	99.0%
N_029	100000000	84119900	10069.2	106.0	0	2050	99.0%
N_030	88000000	75626602	7989.7	84.0	0	1923	99.0%
N_031	89800000	76411742	7220.4	77.0	0	1833	99.0%
N_032	85200000	72704462	8214.2	87.0	0	1686	99.0%
N_033	102600000	73471438	9152.0	93.0	0	1627	99.0%
N_034	115400000	80027658	9446.0	96.0	0	1909	99.0%
N_035	121000000	82073610	10143.9	104.0	0	2042	99.0%
N_036	99600000	70473708	9564.3	98.0	0	1709	99.0%
N_037	153400000	100333288	8736.7	89.0	0	2575	99.0%
N_038	114400000	79662948	8390.8	87.0	0	1877	99.0%
N_039	101200000	71968384	7971.6	82.0	0	1769	99.0%
N_040	120400000	81830532	9206.5	94.0	0	1933	99.0%
N_041	134000000	91081266	7602.9	76.0	0	2328	99.0%
N_042	138000000	93996252	7225.9	72.0	0	2520	99.0%
N_043	154600000	100914114	7768.1	77.0	0	2403	99.0%
N_044	140600000	95170456	7855.7	77.0	0	2292	99.0%
N_045	127200000	86975448	8774.5	88.0	0	1988	99.0%
N_046	116400000	83567574	8183.3	81.0	0	2001	99.0%
N_047	111000000	79298270	7533.8	75.0	0	1817	99.0%
N_048	133000000	91621982	7997.0	80.0	0	2377	99.0%
N_049	108800000	91529320	8628.7	85.0	0	2373	99.0%
N_050	101800000	85892198	8441.9	83.0	0	2067	99.0%
N_051	114600000	95458088	9656.8	96.0	0	2454	99.0%
N_052	110800000	93078508	8959.2	89.0	0	2080	99.0%
N_053	88600000	75664626	8927.1	88.0	0	1788	99.0%
N_054	106200000	89523476	9465.8	94.0	0	1928	99.0%
N_055	84400000	72372718	7219.5	71.0	0	1701	99.0%
N_056	94200000	80227240	8538.3	84.0	0	2224	99.0%
N_057	92800000	79688898	9476.0	99.0	0	1736	99.0%
N_058	97600000	82872738	7987.6	84.0	0	2076	99.0%
N_059	112200000	94508806	9035.8	96.0	0	2292	99.0%
N_060	119800000	99865990	7249.2	76.0	0	2490	99.0%
N_061	110600000	91512622	8418.9	89.0	0	2415	99.0%

N_062	85400000	73428638	7573.6	79.0	0	1912	99.0%
N_063	109400000	91479158	7641.4	81.0	0	2402	99.0%
N_064	89200000	76276228	7263.2	77.0	0	1720	99.0%
N_065	104600000	75727686	9174.2	94.0	0	1883	99.0%
N_066	101000000	71991364	8607.1	87.0	0	1891	99.0%
N_067	109000000	77367076	9561.6	98.0	0	1876	99.0%
N_068	111200000	78217332	9325.0	95.0	0	1799	99.0%
N_069	129400000	87364950	7585.8	77.0	0	2196	99.0%
N_070	116200000	81610044	8972.8	90.0	0	1933	99.0%
N_071	105800000	75028444	7250.2	74.0	0	1772	99.0%
N_072	112400000	79687240	8036.1	81.0	0	2062	99.0%
N_073	123200000	85907714	7985.4	75.0	0	2206	99.0%
N_074	119000000	84053078	8306.0	79.0	0	2095	99.0%
N_075	139600000	96152396	9469.9	90.0	0	2456	99.0%
N_076	131600000	89221870	10005.3	96.0	0	2286	99.0%
N_077	127600000	88927116	9174.2	87.0	0	2070	99.0%
N_078	136600000	94340742	7352.3	70.0	0	2410	98.0%
N_079	100800000	71907044	9169.3	85.0	0	1743	99.0%
N_080	124400000	85078162	7642.9	72.0	0	2025	99.0%
N_081	99200000	84385714	8447.7	85.0	0	2027	99.0%
N_082	115800000	96872590	9680.4	99.0	0	2307	99.0%
N_083	114000000	96598268	9676.8	97.0	0	2392	99.0%
N_084	110200000	93180314	9329.3	94.0	0	1979	99.0%
N_085	104400000	88409020	8848.6	88.0	0	2103	99.0%
N_086	85600000	72936470	7305.2	74.0	0	1872	99.0%
N_087	106000000	90173836	9030.6	90.0	0	2232	99.0%
N_088	109800000	92587178	9279.5	94.0	0	2197	99.0%
N_089	104000000	86378168	8638.3	87.0	0	2226	99.0%
N_090	104200000	87480814	8741.4	88.0	0	2209	99.0%
N_091	102600000	85605428	8558.1	86.0	0	2246	99.0%
N_092	113800000	94820612	9475.8	95.0	0	2452	99.0%
N_093	80000000	69006124	6893.6	69.0	0	1676	98.0%
N_094	94400000	79796840	7967.8	80.0	0	2383	99.0%
N_095	94000000	79430004	7933.6	80.0	0	2176	99.0%
N_096	88000000	75044702	7497.4	76.0	0	1744	99.0%

Supplementary Table 4. Distribution of available IHC biomarker staining for patients according to the radiomic clusters.

**Variables**	**Cluster 1 n (%)**	**Cluster 2 n (%)**	**Cluster 3 n (%)**	***P-value*[a]**
**P53 (n=88)**				**0.04**
Wild-type (SI 1-3)	21 (68)	21 (70)	10 (37)	
Negative (SI 0)	3 (10)	0 (0)	4 (15)	
Over expression (SI 4-9)	7 (23)	9 (30)	13 (48)	
**PD-L1 (n=92)**				0.98
Low (SI 0-3)	25 (76)	24 (77)	21 (75)	
High (SI 4-9)	8 (24)	7 (23)	7 (25)	
**HLA-DQB1 tumor and stroma (n=84)**				0.8
Low (Score 0-6)	9 (30)	11 (37)	7 (29)	
High (Score 9-18)	21 (70)	19 (63)	17 (71)	
**LIMCH1 (n=87)**				0.11/0.06[c]
Low (SI 0-3)	7 (24)	6 (19)	1 (4)	
High (SI 4-9)	22 (76)	26 (81)	25 (96)	

a: Chi-square test

b: Kruskal-Wallis Test

c: Fisher's Exact Test, Cluster 1/2 versus 3

Missing info: Grade, n=9

Abbreviations: CC: Cervical cancer, FIGO: International Federation of Gynecology and Obstetrics, BSO: Bilateral salpingo-oophorectomy: Lymphadenectomy

Supplementary Table 5a: Kruskal-Wallis test statistics for MRI sequence and feature group feature importance ranking within all radiomic features, Interval I, Interval II and Interval I and II. Only statistically significant finding are reported.

Ranking	Ranking of MRI sequence	Ranking of feature groups
Overall ranking (all 293 radiomic features)	No significant difference	No significant difference
Interval I; Ranking 1-8	Only T2 sequences included	Kruskal-Wallis, p>0.07
Interval II; Ranking 9-55	T2 vs ADC Kruskal-Wallis, p=0.001	No significant difference
Interval I and II; Ranking 1-55	T2 vs ADC Kruskal-Wallis, p<0.001	No significant difference

Supplementary Table 5b: Comparison of mean value of all radiomic features within each cluster ordered based on highest mean inter-difference between clusters. The horizontal lines indicate the ranges of Interval I and II.

Ranking	Variable Name	Cluster 1	Cluster 2	Cluster 3	P-values
1	glrlmRunPercentageT2	0.278	0.485	-1.081	7.91E-16
2	gldmLargeDependenceEmphasisT2	-0.264	-0.496	1.075	1.09E-15
3	glrlmRunLengthNonUniformityNormalizedT2	0.276	0.479	-1.070	2.11E-15
4	glrlmShortRunEmphasisT2	0.250	0.504	-1.064	2.11E-15
5	gldmDependenceVarianceT2	-0.263	-0.450	1.012	1.98E-13
6	glcmIdmT2	-0.119	-0.572	0.956	4.05E-13
7	glcmIdT2	-0.115	-0.574	0.952	4.52E-13
8	glcmDifferenceEntropyT2	0.060	0.606	-0.911	1.31E-12
9	glrlmRunPercentageDWI	0.755	-0.396	-0.619	3.81E-14
10	firstorderUniformityT2	0.106	-0.692	0.774	6.95E-12
11	glcmJointEntropyT2	-0.109	0.694	-0.772	6.93E-12
12	firstorderEntropyT2	-0.134	0.708	-0.753	5.77E-12
13	glrlmRunLengthNonUniformityNormalizedDWI	0.740	-0.379	-0.619	1.51E-13
14	glcmDifferenceAverageT2	0.098	0.536	-0.875	7.29E-11
15	glrlmGrayLevelNonUniformityNormalizedT2	0.139	-0.696	0.730	2.28E-11
16	glcmIdnADC	-0.506	-0.084	0.888	4.36E-11
17	gldmDependenceNonUniformityNormalizedT2	0.271	0.367	-0.911	2.13E-10
18	firstorderMeanAbsoluteDeviationT2	-0.105	0.667	-0.741	7.73E-11
19	gldmSmallDependenceEmphasisDWI	0.740	-0.490	-0.469	3.70E-13
20	glszmZonePercentageDWI	0.738	-0.486	-0.471	4.49E-13
21	gldmLargeDependenceEmphasisDWI	-0.697	0.311	0.645	3.76E-12
22	firstorderRobustMeanAbsoluteDeviationT2	-0.069	0.636	-0.755	2.19E-10
23	gldmDependenceVarianceDWI	-0.646	0.191	0.730	2.08E-11
24	glcmMaximumProbabilityT2	0.040	-0.612	0.767	4.03E-10
25	glrlmShortRunEmphasisDWI	0.717	-0.425	-0.522	2.72E-12
26	gldmDependenceNonUniformityNormalizedDWI	0.691	-0.327	-0.615	9.60E-12
27	glcmJointEnergyT2	0.081	-0.635	0.734	4.40E-10
28	firstorderInterquartileRangeT2	-0.075	0.629	-0.737	5.22E-10
29	glrlmRunPercentageADC	0.081	0.513	-0.818	1.72E-09
30	glszmSizeZoneNonUniformityNormalizedDWI	0.713	-0.550	-0.346	2.56E-12
31	glcmSumEntropyT2	-0.207	0.698	-0.628	2.45E-10
32	gldmSmallDependenceEmphasisT2	0.263	0.342	-0.866	2.74E-09
33	gldmLargeDependenceEmphasisADC	-0.146	-0.451	0.834	3.58E-09
34	ngtdmContrastT2	0.233	0.367	-0.853	4.38E-09
35	glcmDifferenceEntropyADC	-0.284	0.720	-0.539	2.67E-10
36	glrlmRunLengthNonUniformityNormalizedADC	0.043	0.532	-0.785	3.73E-09
37	glcmIdDWI	-0.687	0.399	0.510	3.78E-11
38	glcmInverseVarianceT2	-0.148	-0.437	0.818	9.28E-09
39	glszmSmallAreaEmphasisDWI	0.682	-0.547	-0.303	2.89E-11
40	glcmIdmDWI	-0.675	0.391	0.503	9.82E-11
41	gldmDependenceVarianceADC	-0.202	-0.378	0.821	1.87E-08
42	glszmZonePercentageT2	0.268	0.306	-0.825	2.40E-08
43	glcmIdADC	0.090	-0.601	0.676	9.98E-09

44	glcmDifferenceEntropyDWI	0.671	-0.445	-0.424	1.69E-10
45	glrlmLongRunEmphasisT2	-0.114	-0.450	0.783	2.98E-08
46	firstorderKurtosisADC	-0.500	0.001	0.764	8.03E-09
47	glcmIdmADC	0.077	-0.586	0.675	1.84E-08
48	glcmDifferenceAverageADC	-0.211	0.658	-0.567	6.68E-09
49	firstorder90PercentileADC	-0.427	0.710	-0.307	1.11E-09
50	glcmSumAverageADC	-0.572	0.670	-0.032	3.10E-10
51	glcmJointAverageADC	-0.572	0.670	-0.032	3.10E-10
52	glcmJointEntropyADC	-0.385	0.698	-0.355	2.95E-09
53	glcmDifferenceAverageDWI	0.640	-0.385	-0.459	1.61E-09
54	glrlmShortRunEmphasisADC	-0.025	0.537	-0.689	7.29E-08
55	gldmLargeDependenceHighGrayLevelEmphasisDWI	-0.353	-0.181	0.785	1.02E-07
56	firstorderRootMeanSquaredADC	-0.466	0.694	-0.227	1.75E-09
57	glszmZoneEntropyADC	-0.626	0.329	0.513	3.19E-09
58	firstorderVarianceT2	-0.105	0.585	-0.631	5.79E-08
59	gldmGrayLevelVarianceT2	-0.104	0.584	-0.631	5.94E-08
60	glrlmRunVarianceT2	-0.111	-0.427	0.748	1.78E-07
61	firstorderMeanADC	-0.465	0.684	-0.214	3.05E-09
62	glcmSumSquaresT2	-0.116	0.586	-0.615	8.01E-08
63	glrlmRunEntropyADC	-0.631	0.496	0.294	2.03E-09
64	ngtdmBusynessT2	-0.444	-0.038	0.730	1.42E-07
65	glcmContrastT2	0.061	0.459	-0.713	3.11E-07
66	glrlmGrayLevelVarianceT2	-0.117	0.576	-0.600	1.57E-07
67	firstorderEntropyADC	-0.407	0.671	-0.286	1.34E-08
68	firstorderMedianADC	-0.459	0.665	-0.198	9.60E-09
69	glrlmShortRunLowGrayLevelEmphasisADC	0.605	-0.353	-0.448	1.83E-08
70	glcmIdnT2	-0.420	-0.052	0.712	4.61E-07
71	firstorderRangeADC	-0.569	0.577	0.091	1.08E-08
72	glcmSumEntropyADC	-0.424	0.653	-0.235	3.08E-08
73	glszmLargeAreaEmphasisT2	-0.342	-0.153	0.731	9.45E-07
74	gldmLargeDependenceHighGrayLevelEmphasisADC	-0.587	0.309	0.479	4.85E-08
75	glszmZoneVarianceT2	-0.343	-0.153	0.730	9.71E-07
76	glcmClusterTendencyT2	-0.165	0.584	-0.538	3.61E-07
77	glcmIdmnADC	-0.380	-0.101	0.718	8.44E-07
78	firstorderMaximumADC	-0.540	0.594	0.023	2.09E-08
79	ngtdmStrengthDWI	0.575	-0.289	-0.488	8.99E-08
80	glszmGrayLevelNonUniformityNormalizedADC	0.589	-0.460	-0.280	3.90E-08
81	glrlmRunVarianceDWI	-0.590	0.404	0.355	5.61E-08
82	gldmDependenceEntropyADC	-0.581	0.482	0.236	4.83E-08
83	glszmLargeAreaLowGrayLevelEmphasisT2	-0.280	-0.211	0.713	3.09E-06
84	gldmDependenceNonUniformityNormalizedADC	0.121	0.371	-0.688	3.48E-06
85	glcmInverseVarianceDWI	-0.553	0.254	0.503	2.52E-07
86	glcmDifferenceVarianceT2	0.022	0.451	-0.643	2.98E-06
87	firstorder90PercentileT2	0.017	0.453	-0.640	3.07E-06
88	gldmGrayLevelNonUniformityT2	-0.425	-0.009	0.661	1.85E-06
89	gldmLargeDependenceHighGrayLevelEmphasisT2	-0.350	-0.110	0.685	4.13E-06
90	firstorderMeanAbsoluteDeviationADC	-0.307	0.608	-0.353	5.94E-07
91	gldmSmallDependenceEmphasisADC	-0.169	0.557	-0.495	2.04E-06
92	glrlmLowGrayLevelRunEmphasisADC	0.566	-0.382	-0.348	2.49E-07
93	glrlmLongRunEmphasisDWI	-0.566	0.411	0.309	2.26E-07
94	glcmContrastADC	-0.254	0.588	-0.408	1.22E-06
95	glcmContrastDWI	0.557	-0.357	-0.369	4.33E-07
96	glrlmLongRunHighGrayLevelEmphasisADC	-0.555	0.392	0.318	4.57E-07
97	firstorderRobustMeanAbsoluteDeviationADC	-0.224	0.568	-0.426	2.74E-06
98	glszmGrayLevelNonUniformityNormalizedT2	0.296	-0.582	0.335	2.22E-06
99	ngtdmContrastDWI	0.540	-0.341	-0.364	1.16E-06
100	glszmGrayLevelNonUniformityADC	-0.543	0.391	0.301	9.08E-07
101	firstorder90PercentileDWI	0.492	-0.526	-0.041	9.15E-07
102	glszmZonePercentageADC	-0.163	0.528	-0.464	9.41E-06
103	glcmIdmnT2	-0.367	-0.050	0.628	1.68E-05
104	firstorderInterquartileRangeADC	-0.203	0.543	-0.425	7.66E-06
105	glcmDifferenceVarianceDWI	0.533	-0.389	-0.290	1.51E-06
106	ngtdmContrastADC	-0.026	0.446	-0.564	2.30E-05
107	glrlmGrayLevelNonUniformityT2	-0.416	0.033	0.592	1.33E-05
108	firstorderRootMeanSquaredDWI	0.445	-0.550	0.063	1.73E-06
109	gldmLowGrayLevelEmphasisADC	0.527	-0.387	-0.283	2.03E-06
110	glcmIdnDWI	-0.455	0.116	0.539	1.08E-05
111	shapeMaximum2DDiameterSlice	-0.489	0.197	0.481	6.82E-06
112	glrlmHighGrayLevelRunEmphasisADC	-0.385	0.562	-0.171	3.35E-06
113	gldmHighGrayLevelEmphasisADC	-0.382	0.562	-0.176	3.55E-06

114	firstorder10PercentileADC	-0.443	0.540	-0.054	2.53E-06
115	shapeMinorAxisLength	-0.496	0.227	0.451	6.77E-06
116	shapeLeastAxisLength	-0.304	-0.121	0.629	4.23E-05
117	firstorderMeanDWI	0.424	-0.547	0.091	3.08E-06
118	glrlmShortRunHighGrayLevelEmphasisADC	-0.359	0.555	-0.203	6.02E-06
119	firstorderUniformityADC	0.293	-0.551	0.298	9.77E-06
120	gldmSmallDependenceHighGrayLevelEmphasisT2	0.086	0.345	-0.598	7.29E-05
121	shapeMaximum2DDiameterRow	-0.512	0.352	0.307	5.12E-06
122	glrlmGrayLevelNonUniformityNormalizedADC	0.389	-0.544	0.142	6.53E-06
123	shapeMaximum3DDiameter	-0.507	0.350	0.301	6.79E-06
124	glrlmRunEntropyT2	-0.409	0.534	-0.097	6.53E-06
125	shapeMaximum2DDiameterColumn	-0.503	0.348	0.298	8.33E-06
126	gldmLargeDependenceLowGrayLevelEmphasisT2	0.033	-0.423	0.522	9.45E-05
127	shapeSurfaceVolumeRatio	0.223	0.196	-0.606	0.0001392
128	glcmAutocorrelationADC	-0.367	0.528	-0.153	1.61E-05
129	glcmMaximumProbabilityADC	0.143	-0.480	0.430	7.45E-05
130	glcmDifferenceVarianceADC	-0.287	0.525	-0.271	3.09E-05
131	gldmDependenceEntropyT2	-0.462	0.460	0.084	1.24E-05
132	glszmZoneEntropyT2	-0.458	0.464	0.072	1.29E-05
133	firstorderEntropyDWI	0.447	-0.477	-0.038	1.32E-05
134	glcmCorrelationT2	-0.443	0.159	0.462	4.98E-05
135	firstorderRootMeanSquaredT2	0.045	0.355	-0.550	0.0001998
136	firstorderMedianDWI	0.332	-0.522	0.198	2.88E-05
137	ngtdmStrengthT2	0.314	0.070	-0.574	0.0001645
138	glszmHighGrayLevelZoneEmphasisADC	-0.378	0.512	-0.115	2.35E-05
139	shapeSphericity	0.449	-0.464	-0.059	1.73E-05
140	glszmSizeZoneNonUniformityADC	-0.450	0.458	0.069	1.86E-05
141	glcmJointEntropyDWI	0.460	-0.440	-0.108	1.91E-05
142	glrlmLongRunHighGrayLevelEmphasisDWI	-0.118	-0.287	0.570	0.0002876
143	glcmJointEnergyADC	0.169	-0.479	0.390	0.0001115
144	glcmSumAverageDWI	0.266	-0.501	0.270	8.65E-05
145	glcmJointAverageDWI	0.266	-0.501	0.270	8.65E-05
146	glszmSmallAreaEmphasisADC	-0.438	0.446	0.067	3.49E-05
147	glszmSizeZoneNonUniformityNormalizedADC	-0.410	0.474	-0.015	3.85E-05
148	shapeMajorAxisLength	-0.467	0.342	0.251	4.48E-05
149	glszmLowGrayLevelZoneEmphasisADC	0.458	-0.262	-0.347	6.56E-05
150	gldmSmallDependenceLowGrayLevelEmphasisADC	0.426	-0.160	-0.435	0.0001217
151	glszmGrayLevelNonUniformityT2	-0.431	0.175	0.423	0.0001182
152	glcmCorrelationDWI	-0.319	-0.039	0.541	0.000328
153	firstorderMeanAbsoluteDeviationDWI	0.437	-0.435	-0.081	4.42E-05
154	gldmGrayLevelVarianceADC	-0.290	0.496	-0.227	9.82E-05
155	firstorderVarianceADC	-0.291	0.496	-0.227	9.80E-05
156	glszmGrayLevelVarianceT2	-0.161	0.459	-0.375	0.0002473
157	firstorderMeanT2	0.061	0.319	-0.525	0.0005967
158	glcmIdmnDWI	-0.394	0.115	0.447	0.0002969
159	firstorderRobustMeanAbsoluteDeviationDWI	0.422	-0.420	-0.078	9.13E-05
160	glcmSumEntropyDWI	0.397	-0.448	0.000	0.0001003
161	firstorderInterquartileRangeDWI	0.421	-0.416	-0.081	0.0001008
162	glszmGrayLevelNonUniformityDWI	-0.398	0.133	0.429	0.0003277
163	glszmSmallAreaHighGrayLevelEmphasisADC	-0.337	0.470	-0.120	0.0001633
164	glcmInverseVarianceADC	0.121	-0.426	0.391	0.000578
165	firstorderSkewnessDWI	0.238	0.124	-0.532	0.0010074
166	glszmZoneEntropyDWI	-0.278	-0.071	0.522	0.0008842
167	glrlmRunVarianceADC	-0.116	-0.256	0.523	0.001193
168	gldmDependenceEntropyDWI	-0.141	-0.229	0.525	0.0013156
169	firstorderEnergyADC	-0.418	0.383	0.121	0.0001836
170	firstorderMaximumDWI	0.392	-0.420	-0.031	0.0002095
171	gldmSmallDependenceHighGrayLevelEmphasisDWI	0.419	-0.368	-0.143	0.0002219
172	firstorderRangeT2	-0.388	0.420	0.025	0.0002278
173	gldmSmallDependenceLowGrayLevelEmphasisDWI	0.291	0.040	-0.499	0.0011979
174	glcmSumSquaresADC	-0.260	0.457	-0.221	0.0004411
175	glrlmLongRunEmphasisADC	-0.071	-0.285	0.494	0.0017466
176	firstorderKurtosisT2	-0.208	-0.146	0.515	0.0017806
177	glszmSmallAreaHighGrayLevelEmphasisDWI	0.390	-0.401	-0.054	0.0003174
178	ngtdmBusynessDWI	-0.400	0.192	0.351	0.0006105
179	firstorderMedianT2	0.081	0.271	-0.490	0.0021975
180	gldmSmallDependenceLowGrayLevelEmphasisT2	0.298	0.019	-0.481	0.0015601
181	glszmGrayLevelNonUniformityNormalizedDWI	-0.358	0.425	-0.027	0.0003889
182	glrlmShortRunHighGrayLevelEmphasisT2	-0.061	0.373	-0.411	0.0016816
183	firstorderMinimumT2	0.256	0.077	-0.496	0.0019892

184	glszmHighGrayLevelZoneEmphasisT2	-0.203	0.435	-0.279	0.0009002
185	firstorderMaximumT2	-0.327	0.438	-0.093	0.0004669
186	glszmGrayLevelVarianceDWI	0.400	-0.365	-0.119	0.0004074
187	firstorderMinimumDWI	0.371	-0.126	-0.397	0.0010312
188	glszmSizeZoneNonUniformityT2	-0.397	0.217	0.314	0.0007865
189	gldmSmallDependenceHighGrayLevelEmphasisADC	-0.226	0.432	-0.238	0.0010809
190	ngtdmComplexityADC	-0.274	0.433	-0.167	0.000898
191	ngtdmComplexityDWI	0.399	-0.322	-0.175	0.0006394
192	glrlmGrayLevelVarianceDWI	0.381	-0.372	-0.080	0.0006292
193	glcmSumSquaresDWI	0.385	-0.363	-0.098	0.0006465
194	glszmGrayLevelVarianceADC	-0.333	0.413	-0.050	0.0008079
195	gldmGrayLevelVarianceDWI	0.377	-0.371	-0.074	0.0007205
196	firstorderVarianceDWI	0.377	-0.371	-0.074	0.0007209
197	glrlmShortRunHighGrayLevelEmphasisDWI	0.299	-0.422	0.113	0.0010117
198	glcmClusterTendencyADC	-0.242	0.423	-0.202	0.0014192
199	firstorderTotalEnergyADC	-0.380	0.353	0.104	0.0008424
200	gldmHighGrayLevelEmphasisDWI	0.258	-0.424	0.179	0.0013213
201	glcmClusterShadeT2	-0.158	0.402	-0.302	0.0022278
202	glrlmGrayLevelVarianceADC	-0.234	0.421	-0.211	0.0015459
203	glrlmHighGrayLevelRunEmphasisDWI	0.268	-0.422	0.161	0.0013047
204	glszmHighGrayLevelZoneEmphasisDWI	0.332	-0.402	0.036	0.0010467
205	glrlmShortRunLowGrayLevelEmphasisDWI	-0.104	0.375	-0.349	0.0032042
206	glcmAutocorrelationDWI	0.251	-0.417	0.181	0.0016419
207	glrlmRunLengthNonUniformityADC	-0.381	0.318	0.152	0.0011755
208	glrlmGrayLevelNonUniformityNormalizedDWI	-0.317	0.397	-0.052	0.0014886
209	glszmSmallAreaLowGrayLevelEmphasisADC	0.374	-0.235	-0.254	0.0020391
210	glrlmRunEntropyDWI	0.157	-0.385	0.280	0.0039233
211	glrlmLongRunLowGrayLevelEmphasisT2	0.117	-0.369	0.320	0.0047092
212	firstorderUniformityDWI	-0.312	0.387	-0.048	0.0020004
213	glrlmLowGrayLevelRunEmphasisDWI	-0.141	0.376	-0.293	0.0046824
214	glcmClusterProminenceT2	-0.106	0.359	-0.324	0.0057076
215	glszmSmallAreaHighGrayLevelEmphasisT2	-0.161	0.380	-0.268	0.0047979
216	glcmClusterTendencyDWI	0.344	-0.347	-0.057	0.0022729
217	glrlmHighGrayLevelRunEmphasisT2	-0.095	0.345	-0.320	0.0080703
218	firstorderRangeDWI	0.294	-0.370	0.051	0.0037054
219	ngtdmComplexityT2	-0.191	0.376	-0.216	0.0060594
220	glszmSmallAreaLowGrayLevelEmphasisT2	0.350	-0.237	-0.214	0.0045305
221	glrlmGrayLevelNonUniformityDWI	-0.344	0.200	0.256	0.0052425
222	glcmImc1ADC	0.097	0.200	-0.418	0.0148478
223	glcmCorrelationADC	-0.258	-0.002	0.398	0.0108936
224	ngtdmCoarsenessT2	0.327	-0.140	-0.312	0.0067247
225	gldmHighGrayLevelEmphasisT2	-0.083	0.331	-0.321	0.010716
226	gldmLowGrayLevelEmphasisDWI	-0.154	0.361	-0.253	0.0081866
227	firstorderEnergyDWI	-0.318	0.118	0.326	0.007771
228	glszmLowGrayLevelZoneEmphasisT2	0.340	-0.277	-0.145	0.0052452
229	gldmGrayLevelNonUniformityDWI	-0.334	0.185	0.260	0.0070054
230	glcmClusterShadeDWI	0.191	0.089	-0.413	0.0167161
231	firstorder10PercentileDWI	0.260	-0.362	0.092	0.0065327
232	shapeSurfaceArea	-0.331	0.229	0.197	0.0080804
233	firstorderTotalEnergyDWI	-0.311	0.141	0.285	0.0119386
234	gldmDependenceNonUniformityADC	-0.323	0.247	0.160	0.0095518
235	glszmLargeAreaEmphasisDWI	-0.267	0.046	0.347	0.0180563
236	ngtdmStrengthADC	-0.126	0.330	-0.253	0.0169486
237	glcmMCCDWI	-0.169	-0.099	0.393	0.0263843
238	glszmZoneVarianceDWI	-0.265	0.044	0.345	0.0192827
239	glszmLargeAreaLowGrayLevelEmphasisDWI	-0.242	0.008	0.358	0.02355
240	glcmMCCADC	-0.273	0.066	0.328	0.0198409
241	glcmMaximumProbabilityDWI	-0.274	0.324	-0.019	0.011521
242	glrlmLongRunLowGrayLevelEmphasisDWI	-0.300	0.281	0.078	0.0130696
243	ngtdmCoarsenessADC	0.292	-0.125	-0.277	0.0195599
244	glcmMCCT2	-0.301	0.162	0.240	0.0183252
245	glrlmRunLengthNonUniformityDWI	-0.306	0.211	0.183	0.0168082
246	shapeFlatness	0.121	-0.310	0.234	0.0282244
247	gldmDependenceNonUniformityDWI	-0.302	0.199	0.192	0.0189359
248	glcmAutocorrelationT2	-0.080	0.290	-0.270	0.0341212
249	glcmJointEnergyDWI	-0.248	0.310	-0.040	0.0203597
250	gldmLargeDependenceLowGrayLevelEmphasisADC	0.172	-0.316	0.165	0.0281323
251	glrlmRunLengthNonUniformityT2	-0.289	0.167	0.216	0.0262112
252	glrlmShortRunLowGrayLevelEmphasisT2	0.279	-0.132	-0.249	0.0297046
253	glszmLargeAreaHighGrayLevelEmphasisT2	-0.224	0.011	0.328	0.0421397

254	glcmSumAverageT2	-0.117	0.298	-0.224	0.0372828
255	glcmJointAverageT2	-0.117	0.298	-0.224	0.0372828
256	glrlmLongRunHighGrayLevelEmphasisT2	-0.266	0.097	0.276	0.0340278
257	gldmDependenceNonUniformityT2	-0.284	0.166	0.211	0.0293809
258	glcmImc2DWI	0.213	-0.308	0.091	0.0287256
259	gldmLargeDependenceLowGrayLevelEmphasisDWI	-0.272	0.262	0.062	0.0272883
260	glszmSizeZoneNonUniformityDWI	-0.152	-0.082	0.343	0.0624745
261	glszmLargeAreaLowGrayLevelEmphasisADC	-0.172	-0.054	0.336	0.0625517
262	glszmSmallAreaLowGrayLevelEmphasisDWI	-0.147	0.295	-0.173	0.0452213
263	glszmLowGrayLevelZoneEmphasisDWI	-0.181	0.294	-0.121	0.0443975
264	ngtdmCoarsenessDWI	0.257	-0.121	-0.229	0.0517562
265	glszmSmallAreaEmphasisT2	-0.057	0.251	-0.253	0.0722152
266	firstorderSkewnessT2	-0.260	0.165	0.174	0.0537685
267	glrlmLowGrayLevelRunEmphasisT2	0.261	-0.199	-0.130	0.0501391
268	glcmClusterProminenceDWI	0.251	-0.236	-0.064	0.0493437
269	glrlmGrayLevelNonUniformityADC	-0.254	0.144	0.194	0.0608717
270	gldmLowGrayLevelEmphasisT2	0.251	-0.187	-0.131	0.0640443
271	glcmImc1T2	0.241	-0.120	-0.206	0.0762231
272	firstorder10PercentileT2	0.097	0.113	-0.302	0.1235316
273	firstorderMinimumADC	0.153	0.044	-0.294	0.1197207
274	gldmGrayLevelNonUniformityADC	-0.229	0.106	0.206	0.0960301
275	shapeElongation	0.119	-0.250	0.157	0.1073915
276	firstorderSkewnessADC	-0.122	-0.074	0.287	0.1486223
277	glcmClusterShadeADC	-0.149	0.252	-0.113	0.1036527
278	firstorderTotalEnergyT2	-0.221	0.220	0.041	0.0887294
279	glszmSizeZoneNonUniformityNormalizedT2	-0.019	0.194	-0.233	0.165649
280	TumorVolume	-0.202	0.202	0.035	0.1318487
281	shapeMeshVolume	-0.202	0.202	0.035	0.1318697
282	glcmImc2T2	-0.198	0.196	0.039	0.1450492
283	glszmLargeAreaHighGrayLevelEmphasisDWI	-0.203	0.115	0.156	0.1672847
284	glcmImc1DWI	0.139	0.023	-0.244	0.2193651
285	glcmClusterProminenceADC	-0.119	0.212	-0.105	0.2047147
286	glrlmLongRunLowGrayLevelEmphasisADC	0.010	-0.163	0.205	0.2672074
287	glszmLargeAreaEmphasisADC	-0.166	0.039	0.201	0.2387555
288	glszmZoneVarianceADC	-0.166	0.041	0.198	0.2430387
289	glcmImc2ADC	-0.179	0.098	0.140	0.251306
290	ngtdmBusynessADC	-0.135	0.001	0.206	0.3053519
291	firstorderEnergyT2	-0.161	0.160	0.029	0.2807998
292	glszmLargeAreaHighGrayLevelEmphasisADC	-0.162	0.097	0.117	0.3250983
293	firstorderKurtosisDWI	-0.080	0.131	-0.055	0.5471284

Supplementary Table 6: Number of highest, intermediate, and lowest ranked features for each cluster within all, the top 8 ranked and the 9 to 55 ranked radiomic features. The number of cases in each group is given followed by percentage for each row in parenthesis.

Absolute mean value	*Cluster 1 (n=52)*	*Cluster 2 (n=46)*	*Cluster 3 (n=50)*	**P-value[a]**
**All features**				
Highest	94 (32)	112 (38)	87 (30)	0.02
Intermediate	87 (30)	104 (35)	102 (35)	
Lowest	112 (38)	77 (26)	104 (35)	
**Rank 1-8**				7.61E-08
Highest	0 (0)	0 (0)	8 (100)	
Intermediate	0 (0)	8 (100)	0 (0)	
Lowest	8 (100)	0 (0)	0 (0)	
**Rank 9-55**				7.61E-08
Highest	13 (28)	7 (15)	27 (57)	
Intermediate	8 (17)	28 (60)	11 (23)	
Lowest	26 (55)	12 (26)	9 (19)	

a: Chi-square test

Supplementary Table 7: Druggable targets within the oncoplot genes (Figure 4a). Data was obtained from the Human Protein Atlas Database (https://www.proteinatlas.org/).

#OncoPlot	HGNC Symbol	Ensembl Gene ID	Genomic Location	TDL
1	*PIK3CA*	ENSG00000121879	chr3:179148114-179240093:+	Tclin
2	*KMT2D*	ENSG00000167548	chr12:49018975-49059774:-	Tbio
3	*KMT2C*	ENSG00000055609	chr7:152134922-152436005:-	Tbio
4	*FAT1*	ENSG00000083857	chr4:186587783-186726722:-	Tbio
5	*EP300*	ENSG00000100393	chr22:41091786-41180077:+	Tchem
6	*RB1*	ENSG00000139687	chr13:48303751-48481986:+	Tchem
7	*PTEN*	ENSG00000171862	chr10:87863113-87971930:+	Tbio
8	*DDX3X*	ENSG00000215301	chrX:41333348-41364472:+	Tchem
9	*RANBP2*	ENSG00000153201	chr2:108719481-108785811:+	Tbio
10	*ERBB3*	ENSG00000065361	chr12:56079857-56103505:+	Tchem
11	*MAPK1*	ENSG00000100030	chr22:21754500-21867680:-	Tchem
12	*CASP8*	ENSG00000064012	chr2:201233443-201287711:+	Tchem
13	*TP63*	ENSG00000073282	chr3:189631416-189897279:+	Tbio
14	*ERBB2*	ENSG00000141736	chr17:39687914-39730426:+	Tclin
15	*CREBBP*	ENSG00000005339	chr16:3725054-3880726:-	Tchem
16	*TP53*	ENSG00000141510	chr17:7661779-7687550:-	Tchem

Abbreviations:
HGNC: HUGO Gene Nomenclature Committee
TDL: Target Development Levels
Tclin: These targets have activities in DrugCentral (ie. approved drugs) with known mechanism of action.
Tchem: These targets have activities in ChEMBL or DrugCentral that satisfy the activity thresholds detailed below. In some cases, targets have been manually migrated to Tchem by human curation based on small molecule activities from other sources.
Tbio: These targets do not have known drug or small molecule activities that satisfy the activity thresholds detailed below AND satisfy one or more of the following criteria: target is above the cutoff criteria for Tdark; target is annotated with a Gene Ontology Molecular Function or Biological Process leaf term(s) with an Experimental Evidence code; target has confirmed OMIM phenotype(s)

Supplementary Table 8A: Significantly enriched ontology gene sets in Cluster 1 versus 2 tumors.

Enriched in C1<br>Rank	Gene Set	Count	ES	NES	Nom Pvalue	FDR (%)
1	GO_INTERFERON_GAMMA_MEDIATED_SIGNALING_PATHWAY	76	0.64	2.76	0	0
2	GO_TERTIARY_GRANULE_MEMBRANE	61	0.65	2.66	0	0
3	GO_RESPONSE_TO_INTERFERON_GAMMA	159	0.54	2.61	0	0
4	GO_ALPHA_BETA_T_CELL_ACTIVATION	114	0.56	2.57	0	0
5	GO_ANTIGEN_PROCESSING_AND_PRESENTATION_OF_ ENDOGENOUS_PEPTIDE_ANTIGEN	13	0.9	2.55	0	0
6	GO_ADAPTIVE_IMMUNE_RESPONSE	327	0.47	2.51	0	0
7	GO_TERTIARY_GRANULE	141	0.53	2.5	0	0
8	GO_SECRETORY_GRANULE_MEMBRANE	236	0.49	2.48	0	0
9	GO_IMMUNOLOGICAL_SYNAPSE	29	0.69	2.42	0	0
10	GO_MHC_PROTEIN_COMPLEX	20	0.76	2.41	0	0
11	GO_RESPONSE_TO_TYPE_I_INTERFERON	87	0.55	2.39	0	0
12	GO_ALPHA_BETA_T_CELL_DIFFERENTIATION	86	0.55	2.39	0	0
13	GO_POSITIVE_REGULATION_OF_LEUKOCYTE_CELL_CELL_ADHESION	188	0.48	2.38	0	0
14	GO_FICOLIN_1_RICH_GRANULE_MEMBRANE	50	0.6	2.37	0	0
15	GO_SPECIFIC_GRANULE_MEMBRANE	76	0.56	2.37	0	0
16	GO_POSITIVE_REGULATION_OF_CELL_ACTIVATION	271	0.46	2.36	0	0
17	GO_LEUKOCYTE_ACTIVATION_INVOLVED_IN_INFLAMMATORY_RESPONSE	33	0.66	2.36	0	0
18	GO_INTERFERON_GAMMA_PRODUCTION	90	0.53	2.34	0	0.01
19	GO_T_CELL_RECEPTOR_COMPLEX	17	0.76	2.34	0	0.01
20	GO_LEUKOCYTE_CELL_CELL_ADHESION	281	0.45	2.33	0	0.01

Enriched in C2<br>Rank	Gene Set	Count	ES	NES	Nom Pvalue	FDR (%)
1	GO_ORGANELLAR_RIBOSOME	56	-0.61	-2.5	0	0
2	GO_DNA_REPLICATION_INDEPENDENT_NUCLEOSOME_ORGANIZATION	34	-0.67	-2.47	0	0
3	GO_DNA_PACKAGING_COMPLEX	56	-0.61	-2.45	0	0
4	GO_TRANSLATIONAL_TERMINATION	71	-0.57	-2.44	0	0
5	GO_PROTEIN_LOCALIZATION_TO_CHROMOSOME_CENTROMERIC_REGION	17	-0.8	-2.41	0	0
6	GO_MITOCHONDRIAL_TRANSLATIONAL_TERMINATION	58	-0.59	-2.39	0	0
7	GO_MITOCHONDRIAL_TRANSLATION	91	-0.53	-2.37	0	0.01
8	GO_CHROMATIN_ASSEMBLY	97	-0.52	-2.35	0	0.02
9	GO_DNA_PACKAGING	133	-0.49	-2.31	0	0.05
10	GO_MITOCHONDRIAL_GENE_EXPRESSION	110	-0.49	-2.29	0	0.07
11	GO_CONDENSED_NUCLEAR_CHROMOSOME_CENTROMERIC_REGION	20	-0.73	-2.27	0	0.07
12	GO_CONDENSED_CHROMOSOME_CENTROMERIC_REGION	91	-0.51	-2.27	0	0.07
13	GO_NUCLEAR_DNA_REPLICATION	50	-0.57	-2.27	0	0.07
14	GO_ORGANELLAR_LARGE_RIBOSOMAL_SUBUNIT	36	-0.62	-2.26	0	0.06
15	GO_CHROMATIN_REMODELING_AT_CENTROMERE	26	-0.67	-2.26	0	0.06
16	GO_CONDENSED_NUCLEAR_CHROMOSOME_KINETOCHORE	13	-0.8	-2.25	0	0.06
17	GO_HISTONE_EXCHANGE	36	-0.62	-2.24	0	0.07
18	GO_DNA_DEPENDENT_DNA_REPLICATION	119	-0.48	-2.21	0	0.14
19	GO_CENTROMERE_COMPLEX_ASSEMBLY	34	-0.61	-2.2	0	0.15
20	GO_PROTEIN_DNA_COMPLEX	135	-0.47	-2.2	0	0.14

Supplementary Table 8B. Significantly enriched ontology gene sets in Cluster 1 versus 3 tumors.

Rank	Gene Set	Count	ES	NES	Nom Pvalue	FDR (%)
**Enriched in C1**						
1	GO_BICARBONATE_TRANSMEMBRANE_TRANSPORTER_ACTIVITY	13	0.81	2.14	0	1.38
2	GO_RESPONSE_TO_CHEMOKINE	85	0.53	2.12	0	1.01
3	GO_SEROTONIN_SECRETION	10	0.84	2.08	0	2
4	GO_EXTRACELLULAR_LIGAND_GATED_ION_CHANNEL_ACTIVITY	62	0.55	2.07	0	1.78
5	GO_NON_MEMBRANE_SPANNING_PROTEIN_TYROSINE_KINASE_ACTIVITY	42	0.57	2.05	0	2.01
6	GO_OLFACTORY_RECEPTOR_ACTIVITY	36	0.6	2.03	0	3
7	GO_REGULATION_OF_ANTIGEN_PROCESSING_AND_PRESENTATION	18	0.67	2.01	0	3.43
8	GO_NEUROTRANSMITTER_RECEPTOR_ACTIVITY	89	0.5	1.99	0	4.11
9	GO_HORMONE_ACTIVITY	92	0.49	1.99	0	3.8
10	GO_ADAPTIVE_IMMUNE_RESPONSE	327	0.43	1.98	0	3.55
11	GO_SENSORY_PERCEPTION_OF_CHEMICAL_STIMULUS	100	0.49	1.98	0	3.37
12	GO_T_CELL_ACTIVATION_INVOLVED_IN_IMMUNE_RESPONSE	83	0.49	1.98	0	3.29
13	GO_G_PROTEIN_COUPLED_RECEPTOR_ACTIVITY	353	0.43	1.97	0	3.41
14	GO_ION_ANTIPORTER_ACTIVITY	40	0.56	1.97	0	3.33
15	GO_SEROTONIN_RECEPTOR_ACTIVITY	23	0.63	1.96	0	3.42
16	GO_PROTEIN_O_LINKED_GLYCOSYLATION	65	0.5	1.96	0	3.57
17	GO_POSITIVE_REGULATION_OF_CD4_POSITIVE_ALPHA_BETA_T_CELL_ACTIVATION	31	0.57	1.96	0	3.43
18	GO_SEROTONIN_TRANSPORT	15	0.71	1.95	0	3.3
19	GO_SENSORY_PERCEPTION_OF_SMELL	54	0.53	1.95	0	3.28
20	GO_INTERLEUKIN_10_PRODUCTION	40	0.55	1.95	0	3.32
**Enriched in C3**						
**Rank**	**Gene Set**	**Count**	**ES**	**NES**	**Nom Pvalue**	**FDR (%)**
1	GO_CORNIFIED_ENVELOPE	35	-0.78	-3.18	0	0
2	GO_ENDOPEPTIDASE_COMPLEX	58	-0.66	-3.05	0	0
3	GO_REGULATION_OF_TRANSCRIPTION_FROM_RNA_POLYMERASE_II_ PROMOTER_IN_RESPONSE_TO_HYPOXIA	73	-0.63	-2.97	0	0
4	GO_CORNIFICATION	79	-0.6	-2.87	0	0
5	GO_PROTEASOME_ACCESSORY_COMPLEX	24	-0.77	-2.85	0	0
6	GO_ANAPHASE_PROMOTING_COMPLEX_DEPENDENT_CATABOLIC_PROCESS	72	-0.58	-2.84	0	0
7	GO_REGULATION_OF_CELLULAR_AMINO_ACID_METABOLIC_PROCESS	56	-0.63	-2.83	0	0
8	GO_KERATINIZATION	91	-0.56	-2.83	0	0
9	GO_PEPTIDASE_COMPLEX	76	-0.58	-2.83	0	0
10	GO_REGULATION_OF_ESTABLISHMENT_OF_PLANAR_POLARITY	92	-0.55	-2.81	0	0
11	GO_SCF_DEPENDENT_PROTEASOMAL_UBIQUITIN_DEPENDENT_ PROTEIN_CATABOLIC_PROCESS	72	-0.59	-2.8	0	0
12	GO_RIBOSOMAL_SUBUNIT	145	-0.49	-2.71	0	0
13	GO_COTRANSLATIONAL_PROTEIN_TARGETING_TO_MEMBRANE	92	-0.54	-2.7	0	0
14	GO_SMALL_RIBOSOMAL_SUBUNIT	60	-0.56	-2.69	0	0
15	GO_STRUCTURAL_CONSTITUENT_OF_RIBOSOME	127	-0.49	-2.69	0	0
16	GO_REGULATION_OF_HEMATOPOIETIC_PROGENITOR_CELL_DIFFERENTIATION	76	-0.55	-2.69	0	0
17	GO_MORPHOGENESIS_OF_A_POLARIZED_EPITHELIUM	116	-0.5	-2.68	0	0
18	GO_SKIN_DEVELOPMENT	249	-0.46	-2.65	0	0
19	GO_REGULATION_OF_STEM_CELL_DIFFERENTIATION	98	-0.52	-2.65	0	0
20	GO_REGULATION_OF_WATER_LOSS_VIA_SKIN	16	-0.82	-2.64	0	0

Supplementary Table 8C: Significantly enriched ontology gene sets in Cluster 2 versus 3 tumors

Rank	Gene Set	Count	ES	NES	Nom Pvalue	FDR (%)
**Enriched in C2**						
1	GO_POSITIVE_REGULATION_OF_HETEROTYPIC_CELL_CELL_ADHESION	16	0.76	2.22	0	0.08
2	GO_SENSORY_PERCEPTION_OF_CHEMICAL_STIMULUS	100	0.5	2.11	0	1.78
3	GO_REGULATION_OF_ICOSANOID_SECRETION	18	0.7	2.06	0	4.29
4	GO_POSITIVE_REGULATION_OF_FATTY_ACID_TRANSPORT	17	0.7	2.06	0	3.31
5	GO_EXCRETION	61	0.52	2.04	0	3.63
6	GO_NEUROTRANSMITTER_RECEPTOR_ACTIVITY	89	0.49	2.04	0	3.17
7	GO_DETECTION_OF_STIMULUS_INVOLVED_IN_SENSORY_PERCEPTION	104	0.49	2.03	0	2.76
8	GO_OLFACTORY_RECEPTOR_ACTIVITY	36	0.57	2.01	0	3.66
9	GO_MAINTENANCE_OF_GASTROINTESTINAL_EPITHELIUM	16	0.7	2.01	0	3.35
10	GO_G_PROTEIN_COUPLED_AMINE_RECEPTOR_ACTIVITY	38	0.56	1.99	0	4.04
11	GO_G_PROTEIN_COUPLED_RECEPTOR_ACTIVITY	353	0.41	1.99	0	3.85
12	GO_EXTRACELLULAR_LIGAND_GATED_ION_CHANNEL_ACTIVITY	62	0.5	1.97	0	4.3
13	GO_SENSORY_PERCEPTION_OF_BITTER_TASTE	25	0.6	1.96	0	4.62
14	GO_VITAMIN_TRANSPORT	29	0.59	1.96	0	4.5
15	GO_DETECTION_OF_CHEMICAL_STIMULUS_INVOLVED_IN_SENSORY_ PERCEPTION_OF_TASTE	23	0.61	1.95	0	4.62
**Enriched in C3**						
**Rank**	**Gene Set**	**Count**	**ES**	**NES**	**Nom Pvalue**	**FDR (%)**
1	GO_COTRANSLATIONAL_PROTEIN_TARGETING_TO_MEMBRANE	92	-0.7	-3.64	0	0
2	GO_CYTOSOLIC_RIBOSOME	95	-0.67	-3.53	0	0
3	GO_TRANSLATIONAL_INITIATION	166	-0.61	-3.46	0	0
4	GO_NUCLEAR_TRANSCRIBED_MRNA_CATABOLIC_ PROCESS_NONSENSE_MEDIATED_DECAY	112	-0.63	-3.38	0	0
5	GO_ESTABLISHMENT_OF_PROTEIN_LOCALIZATION_ TO_ENDOPLASMIC_RETICULUM	101	-0.63	-3.35	0	0
6	GO_PROTEIN_LOCALIZATION_TO_ENDOPLASMIC_RETICULUM	121	-0.59	-3.19	0	0
7	GO_PROTEIN_TARGETING_TO_MEMBRANE	156	-0.56	-3.19	0	0
8	GO_CYTOSOLIC_LARGE_RIBOSOMAL_SUBUNIT	50	-0.69	-3.13	0	0
9	GO_CYTOSOLIC_SMALL_RIBOSOMAL_SUBUNIT	40	-0.72	-3.12	0	0
10	GO_CYTOPLASMIC_TRANSLATION	76	-0.61	-3.03	0	0
11	GO_STRUCTURAL_CONSTITUENT_OF_RIBOSOME	127	-0.54	-3.02	0	0
12	GO_RIBOSOMAL_SUBUNIT	145	-0.53	-2.98	0	0
13	GO_REGULATION_OF_TRANSCRIPTION_FROM_RNA_POLYMERASE_ II_PROMOTER_IN_RESPONSE_TO_HYPOXIA	73	-0.58	-2.85	0	0
14	GO_POLYSOME	59	-0.58	-2.82	0	0
15	GO_ESTABLISHMENT_OF_PROTEIN_LOCALIZATION_TO_MEMBRANE	258	-0.45	-2.81	0	0
16	GO_NUCLEAR_TRANSCRIBED_MRNA_CATABOLIC_PROCESS	170	-0.48	-2.73	0	0
17	GO_CORNIFIED_ENVELOPE	35	-0.64	-2.73	0	0
18	GO_VIRAL_GENE_EXPRESSION	182	-0.46	-2.71	0	0
19	GO_SMALL_RIBOSOMAL_SUBUNIT	60	-0.56	-2.66	0	0
20	GO_POLYSOMAL_RIBOSOME	26	-0.68	-2.65	0	0

Supplementary Table 9A: Significantly enriched Hallmark gene sets in Cluster 1 versus 2 tumors

Rank	Gene Set	Count	ES	NES	Nom Pvalue	FDR (%)
**Enriched in C1**						
1	HALLMARK_INTERFERON_GAMMA_RESPONSE	181	0.65	3.19	0	0
2	HALLMARK_ALLOGRAFT_REJECTION	199	0.63	3.09	0	0
3	HALLMARK_INTERFERON_ALPHA_RESPONSE	82	0.7	3.01	0	0
4	HALLMARK_INFLAMMATORY_RESPONSE	194	0.53	2.65	0	0
5	HALLMARK_TNFA_SIGNALING_VIA_NFKB	197	0.52	2.61	0	0
6	HALLMARK_IL6_JAK_STAT3_SIGNALING	86	0.54	2.36	0	0
7	HALLMARK_COMPLEMENT	186	0.45	2.21	0	0
8	HALLMARK_IL2_STAT5_SIGNALING	176	0.39	1.92	0	0.04
9	HALLMARK_KRAS_SIGNALING_UP	195	0.38	1.86	0	0.08
10	HALLMARK_APOPTOSIS	159	0.34	1.66	0	0.56
11	HALLMARK_P53_PATHWAY	199	0.31	1.52	0	1.74
**Enriched in C2**						
**Rank**	**Gene Set**	**Count**	**ES**	**NES**	**Nom Pvalue**	**FDR (%)**
1	HALLMARK_E2F_TARGETS	187	-0.51	-2.51	0	0
2	HALLMARK_G2M_CHECKPOINT	194	-0.46	-2.28	0	0
3	HALLMARK_FATTY_ACID_METABOLISM	144	-0.42	-2.01	0	0
4	HALLMARK_OXIDATIVE_PHOSPHORYLATION	184	-0.4	-1.94	0	0.07
5	HALLMARK_MYC_TARGETS_V1	198	-0.36	-1.77	0	0.44

Supplementary Table 9B: Significantly enriched gene sets in Cluster 1 versus 3 tumors

Rank	Gene Set	Count	ES	NES	Nom Pvalue	FDR (%)
**Enriched in C1**						
1	HALLMARK_ALLOGRAFT_REJECTION	199	0.49	2.16	0	0
2	HALLMARK_INFLAMMATORY_RESPONSE	194	0.44	1.96	0	0.02
3	HALLMARK_IL6_JAK_STAT3_SIGNALING	86	0.47	1.88	0	0.08
4	HALLMARK_INTERFERON_GAMMA_RESPONSE	181	0.4	1.77	0	0.18
5	HALLMARK_INTERFERON_ALPHA_RESPONSE	82	0.44	1.76	0	0.16
6	HALLMARK_TNFA_SIGNALING_VIA_NFKB	197	0.36	1.61	0	0.9
7	HALLMARK_COMPLEMENT	186	0.34	1.53	0	1.76
8	HALLMARK_KRAS_SIGNALING_DN	191	0.33	1.45	0	3.72
**Enriched in C3**						
**Rank**	**Gene Set**	**Count**	**ES**	**NES**	**Nom Pvalue**	**FDR (%)**
1	HALLMARK_OXIDATIVE_PHOSPHORYLATION	184	-0.51	-2.84	0	0
2	HALLMARK_MYC_TARGETS_V1	198	-0.45	-2.54	0	0
3	HALLMARK_MTORC1_SIGNALING	198	-0.41	-2.34	0	0
4	HALLMARK_EPITHELIAL_MESENCHYMAL_TRANSITION	194	-0.35	-1.99	0	0
5	HALLMARK_FATTY_ACID_METABOLISM	144	-0.36	-1.97	0	0
6	HALLMARK_PROTEIN_SECRETION	94	-0.39	-1.97	0	0
7	HALLMARK_TGF_BETA_SIGNALING	52	-0.38	-1.71	0.01	0.48
8	HALLMARK_CHOLESTEROL_HOMEOSTASIS	61	-0.37	-1.69	0.02	0.42
9	HALLMARK_P53_PATHWAY	199	-0.29	-1.68	0	0.48
10	HALLMARK_ESTROGEN_RESPONSE_LATE	198	-0.29	-1.67	0	0.48
11	HALLMARK_ADIPOGENESIS	174	-0.29	-1.64	0	0.87
12	HALLMARK_GLYCOLYSIS	186	-0.28	-1.56	0	1.87
13	HALLMARK_MITOTIC_SPINDLE	179	-0.27	-1.55	0	1.83
14	HALLMARK_NOTCH_SIGNALING	28	-0.38	-1.47	0.15	3.1
15	HALLMARK_PI3K_AKT_MTOR_SIGNALING	102	-0.28	-1.46	0.01	3.21
16	HALLMARK_ESTROGEN_RESPONSE_EARLY	197	-0.26	-1.46	0.01	3.13

Supplementary Table 9C: Significantly enriched gene sets within Cluster 2 versus 3 tumors.

Rank	Gene Set	Count	ES	NES	Nom Pvalue	FDR (%)
**Enriched in C2**						
1	HALLMARK_E2F_TARGETS	187	0.37	1.71	0	1.2
2	HALLMARK_KRAS_SIGNALING_DN	191	0.35	1.61	0	2.18
3	HALLMARK_SPERMATOGENESIS	125	0.35	1.53	0	3.77
**Enriched in C3**						
**Rank**	**Gene Set**	**Count**	**ES**	**NES**	**Nom Pvalue**	**FDR (%)**
1	HALLMARK_P53_PATHWAY	199	-0.41	-2.38	0	0
2	HALLMARK_OXIDATIVE_PHOSPHORYLATION	184	-0.38	-2.22	0	0
3	HALLMARK_MTORC1_SIGNALING	198	-0.37	-2.2	0	0
4	HALLMARK_PROTEIN_SECRETION	94	-0.42	-2.18	0	0
5	HALLMARK_EPITHELIAL_MESENCHYMAL_TRANSITION	194	-0.36	-2.12	0	0
6	HALLMARK_HYPOXIA	188	-0.36	-2.11	0	0
7	HALLMARK_MYC_TARGETS_V1	198	-0.34	-1.98	0	0
8	HALLMARK_CHOLESTEROL_HOMEOSTASIS	61	-0.4	-1.93	0	0
9	HALLMARK_ANGIOGENESIS	36	-0.41	-1.73	0	0.49
10	HALLMARK_ANDROGEN_RESPONSE	97	-0.32	-1.71	0	0.55
11	HALLMARK_TGF_BETA_SIGNALING	52	-0.36	-1.64	0	0.9
12	HALLMARK_PI3K_AKT_MTOR_SIGNALING	102	-0.31	-1.62	0	1.01
13	HALLMARK_APICAL_JUNCTION	181	-0.27	-1.55	0	1.86
14	HALLMARK_MITOTIC_SPINDLE	179	-0.25	-1.46	0	3.73

Supplementary Note

Reference for explanation of each feature: https://pyradiomics.readthedocs.io/en/latest/

Shape (Only for mask image)

Elongation
Flatness
LeastAxisLength
MajorAxisLength
Maximum2DDiameterColumn
Maximum2DDiameterRow
Maximum2DDiameterSlice
Maximum3DDiameter
MinorAxisLength
Sphericity
SurfaceArea
SurfaceVolumeRatio

The remaining features are 3x multiplied, once for each MR series.

firstorder:

10Percentile
90Percentile
Energy
Entropy
InterquartileRange
Kurtosis
Maximum
Mean
MeanAbsoluteDeviation
Median
Minimum
Range
RobustMeanAbsoluteDeviation
RootMeanSquared
Skewness
TotalEnergy
Uniformity
Variance

glcm:

Autocorrelation
ClusterProminence
ClusterShade
ClusterTendency
Contrast
Correlation
DifferenceAverage
DifferenceEntropy

DifferenceVariance
Id
Idm
Idmn
Idn
Imc1
Imc2
InverseVariance
JointAverage
JointEnergy
JointEntropy
MCC
MaximumProbability
SumAverage
SumEntropy
SumSquares

glszm:
GrayLevelNonUniformity
GrayLevelNonUniformityNormalized
GrayLevelVariance
HighGrayLevelZoneEmphasis
LargeAreaEmphasis
LargeAreaHighGrayLevelEmphasis
LargeAreaLowGrayLevelEmphasis
LowGrayLevelZoneEmphasis
SizeZoneNonUniformity
SizeZoneNonUniformityNormalized
SmallAreaEmphasis
SmallAreaHighGrayLevelEmphasis
SmallAreaLowGrayLevelEmphasis
ZoneEntropy
ZonePercentage
ZoneVariance

nNgtdm
ngtdmBusyness
ngtdmCoarseness
ngtdmComplexity
ngtdmContrast
ngtdmStrength

glrlm
GrayLevelNonUniformity
GrayLevelNonUniformityNormalized
GrayLevelVariance
HighGrayLevelRunEmphasis
LongRunEmphasis
LongRunHighGrayLevelEmphasis
LongRunLowGrayLevelEmphasis
LowGrayLevelRunEmphasis

RunEntropy
RunLengthNonUniformity
RunLengthNonUniformityNormalized
RunPercentage
RunVariance
ShortRunEmphasis
ShortRunHighGrayLevelEmphasis
ShortRunLowGrayLevelEmphasis

gldm:
DependenceEntropy
DependenceNonUniformity
DependenceNonUniformityNormalized
DependenceVariance
GrayLevelNonUniformity
GrayLevelVariance
HighGrayLevelEmphasis
LargeDependenceEmphasis
LargeDependenceHighGrayLevelEmphasis
LargeDependenceLowGrayLevelEmphasis
LowGrayLevelEmphasis
SmallDependenceEmphasis
SmallDependenceHighGrayLevelEmphasis
SmallDependenceLowGrayLevelEmphasis
